# Spatio-temporal Dynamics and Mechanisms of Stress Granule Assembly

**DOI:** 10.1371/journal.pcbi.1004326

**Published:** 2015-06-26

**Authors:** Daisuke Ohshima, Kyoko Arimoto-Matsuzaki, Taichiro Tomida, Mutsuhiro Takekawa, Kazuhisa Ichikawa

**Affiliations:** 1 Division of Mathematical Oncology, The Institute of Medical Science, The University of Tokyo, Tokyo, Japan; 2 Division of Molecular Cell Signaling, The Institute of Medical Science, The University of Tokyo, Tokyo, Japan; 3 Division of Cell Signaling and Molecular Medicine, The Institute of Medical Science, The University of Tokyo, Tokyo, Japan; Johns Hopkins University, UNITED STATES

## Abstract

Stress granules (SGs) are non-membranous cytoplasmic aggregates of mRNAs and related proteins, assembled in response to environmental stresses such as heat shock, hypoxia, endoplasmic reticulum (ER) stress, chemicals (e.g. arsenite), and viral infections. SGs are hypothesized as a loci of mRNA triage and/or maintenance of proper translation capacity ratio to the pool of mRNAs. In brain ischemia, hippocampal CA3 neurons, which are resilient to ischemia, assemble SGs. In contrast, CA1 neurons, which are vulnerable to ischemia, do not assemble SGs. These results suggest a critical role SG plays in regards to cell fate decisions. Thus SG assembly along with its dynamics should determine the cell fate. However, the process that exactly determines the SG assembly dynamics is largely unknown. In this paper, analyses of experimental data and computer simulations were used to approach this problem. SGs were assembled as a result of applying arsenite to HeLa cells. The number of SGs increased after a short latent period, reached a maximum, then decreased during the application of arsenite. At the same time, the size of SGs grew larger and became localized at the perinuclear region. A minimal mathematical model was constructed, and stochastic simulations were run to test the modeling. Since SGs are discrete entities as there are only several tens of them in a cell, commonly used deterministic simulations could not be employed. The stochastic simulations replicated observed dynamics of SG assembly. In addition, these stochastic simulations predicted a gamma distribution relative to the size of SGs. This same distribution was also found in our experimental data suggesting the existence of multiple fusion steps in the SG assembly. Furthermore, we found that the initial steps in the SG assembly process and microtubules were critical to the dynamics. Thus our experiments and stochastic simulations presented a possible mechanism regulating SG assembly.

## Author Summary

Cells suffer from various environmental stresses such as heat shock and viral infection. In response to a stress, small non-membranous cytoplasmic aggregates, stress granules (SGs), are assembled. SGs contain mRNAs and related proteins. Hippocampal CA1 neurons located in the brain, which are vulnerable to ischemia, do not assemble SGs, while CA3 neurons, which are resilient to ischemia, assemble SGs. The dysfunction of SGs has been reported in human diseases including pathogenic viral infection. These observations led to a hypothesis that SGs play an important role in cell fate decisions, and the dynamics of SG assembly would regulate cell fate. However, the conditions that determine the number and distribution of SGs in a cell in response to a stress are largely unknown. We approached this problem by experiments and simulations. Our stochastic simulations replicated the observations. Furthermore, we found that initial steps in the SG assembly process were important to the dynamics of SG assembly, and that SG size resembled the gamma distribution both in simulations and experiments, suggesting the existence of multiple steps in the SG assembly process. To the best of our knowledge, this work was the first to show SG assembly in a whole cell by stochastic simulations.

## Introduction

Cells suffer from various environmental stresses including heat shock, chemicals, hypoxia, starvation, osmotic shock, ultraviolet irradiation, and viral infections. Cells respond to these stresses resulting in either survival or apoptosis. Assembly of stress granules (SGs), which are non-membranous cytoplasmic aggregates of mRNAs and related proteins with a size in the order of 0.1–2 μm, is one form of cellular response to a stress [1–5]. SGs are reported to contain RNA-binding proteins (e.g. HuR), translation initiation factors (e.g. eIF4E, eIF4G, eIF3, and PABP-1), 40S ribosomal subunit, self-oligomerizing proteins (e.g. TIA-1 and G3BP), nuclear transport proteins (e.g. importin α1 and importin 8), and signaling proteins (e.g. TRAF2, RACK1, and Raptor) in addition to mRNAs [2,5–7]. The 60S ribosomal subunit, HSP90 and ARE-binding proteins hnRNPA1 and hnRNPD are excluded from SGs. Inclusion of the translation initiation factor eIF2α and heat shock protein HSP70 are reported to be cell-type and stress-type specific [8]. S1 Fig shows the translation initiation steps (thin lines and narrow characters) together with pathways related to SG assembly (thick lines and bold characters). It has been reported that the SG assembly is usually initiated by the phosphorylation of eIF2α on Ser51 [1,8–10]. This phosphorylation inhibits translation initiation by reducing the level of eIF2 ∙ GTP ∙ tRNA^Met^ ternary complex [1,11]. The observations led to the hypothesis that SGs act as sites for storing and/or sorting of untranslated mRNAs [1,4,12,13]. It has also been hypothesized that SGs maintain the proper ratio of translation capacity to the pool of mRNAs in response to environmental stress [14,15]. In fact, global translation repression is not required for the assembly of SGs [16,17]. In addition, exclusion of mRNAs encoding HSP70 and HSP90 from SGs [1,2,18,19] is consistent with these hypotheses, because this enables HSP70 and HSP90 proteins translated under a stress condition to act as chaperones regulating misfolded proteins outside SGs. Thus, the assembly of SGs offers a chance for a cell to decide its own fate.

The roles of SGs are typically found in brain ischemia. The ischemic treatment of neurons located in the hippocampal CA3 region led to the phosphorylation of eIF2α resulting in the inhibition of protein synthesis [20,21]. Reperfusion with a normal oxygen content solution recovered the protein synthesis in these neurons. However, the recovery did not occur in pyramidal neurons within the hippocampal CA1 region [21–23]. In parallel, the SG assembly was observed in hippocampal CA3 neurons but not in CA1 neurons [20,21]. It is known that the CA1 region is more vulnerable to ischemia than the CA3 region [24,25]. The fact that no SG is assembled in hippocampal CA1 pyramidal neurons by ischemic treatment is a possible explanation for their vulnerability highlighting the role of SGs described above. In addition, SGs are emerging to play an important role in neurodegenerative disorders including ALS (amyotrophic lateral sclerosis) and FTLD (frontotemporal lobar degeneration) [20]. These observations support a view that SGs play an important role in the survival of neurons.

TIA-1 plays a critical role in the induction of SG assembly (S1 Fig). TIA-1 contains one prion-related domain (PRD) and three RNA recognition motifs (RRM1, RRM2, and RRM3) [6,26]. PRD enables TIA-1 to self-aggregate, while RRM2 and RRM3 bind mRNAs with a different nucleic acid sequence specificity [1,6,12,18,27–30]. TIA-1 is required for SG assembly because TIA-1 dominant-negative mutants block SG assembly in response to stress. On the other hand, overexpression of TIA-1 was sufficient to induce SG assembly in the absence of a stress [8,29]. TIAR and G3BP are also known to promote aggregate formation in SG assembly [9,17,31]. Phosphorylation of eIF2α is catalyzed by kinases PKR, PERK, GCN2, and HRI by the following stress indicators: PKR by heat shock, UV irradiation, and viral infection; PERK by ER stress; GCN2 by starvation; HRI by hypoxia [32–38]. In addition to the initiation of SG assembly by the phosphorylation of eIF2α, it also promotes polysomal disassembly resulting in the accumulation of untranslated mRNPs [33]. From these observations, it is postulated that self-aggregating proteins such as TIA-1 play a major role in aggregate formation, while eIF2α phosphorylation provides constituents of SGs, thus both acting synergistically in the SG assembly. In addition, it is also postulated that TIA-1 actively escorts untranslated mRNA to SGs [6].

SGs were assembled after a short latent period (<10 min) by the application of arsenite. The number of SGs reached a peak after approximately 20–30 min (~30 SGs), and then subsequently decreased [39]. The physical size of SGs was small early in the assembly, and they maturated into larger size later [37,40,41]. SGs distributed within the cytoplasm without preference to location at the beginning of the assembly, and were gradually confined to the perinuclear region later as a response to arsenite [39]. Microtubules were reported to be critical for SG assembly, because inhibition of their function abrogated SG assembly [41,42]. In addition, SGs were reported to move on microtubules by the dynein motor [37,42].

It is reasonable to assume that the dynamics of SG assembly should be closely related to its biological roles. The time course over which SG assembly occurs is important in determining how quickly the cell can respond to a stressful environment. The number and the size of SGs can be considered as measures of responsiveness of a cell to environmental stress. The distribution of SGs in a cell can indicate the location in a cell where the translational silencing occurs, and where the most effective loci of translational silencing and recovery take place at specific mRNAs. Although there are reports on the dynamics of SGs as shown above, mechanisms that regulate these dynamics are still largely unknown. We approached these problems by devising experiments and conducting computer simulations.

SG simulation is not an extension of conventional deterministic simulation (DS), which is based on differential equations including continuous variables, and concentrations. Since SGs are discrete countable structures, they should be expressed in terms of numbers instead of a concentration. As a result, a stochastic simulation (SS) was employed [43]. In our method of SS, molecules undergo a random walk (RW) changing their location, making chance collisions leading to a reaction described by a probability function *P*_*r*_ calculated from the classical binding reaction rate constant *k*. Thus our SS program controls coordinates and states of every single molecule. Simulation results not only showed good agreements with our observations of SG assembly, but also predicted a gamma distribution relative to SG size. The experimental data also yielded a gamma distribution. In addition, we clarified critical parameters determining the dynamics of SG assembly.

## Results

### Spatio- temporal dynamics of SG assembly in response to aresenite

**Fig 1:**
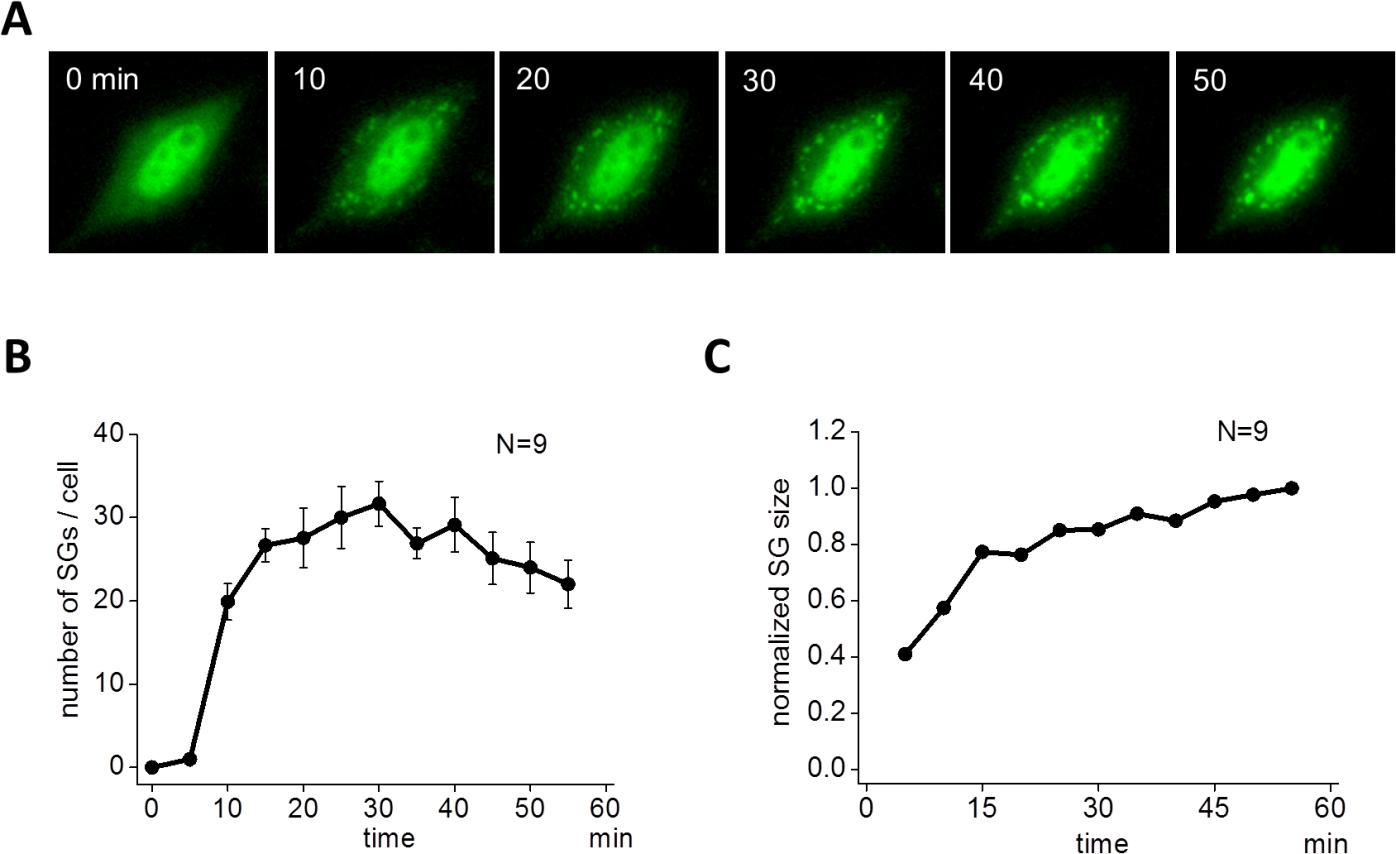
Assembly of SGs by arsenite. (A) SGs were visualized by green fluorescent protein (GFP)-tagged TIA1 in living HeLa cells. Arsenite (0.5 mM) was applied to HeLa cells at 0 min, and SGs were seen as punctate structures after 10 min. SGs gradually localized at perinuclear region. (B) The number of SG increased after a short latency reaching a peak around 30 min, and then it decreased. Data shown is the average of 9 cells. (C) The increase in the average size of SG was sublinear. SG size was averaged for all SGs in 9 cells.

First we investigated the assembly of SGs in HeLa cells. To monitor the process of SG assembly in living cells, HeLa cells were transiently transfected with an expression plasmid for green fluorescent protein (GFP)-tagged TIA-1, an SG-nucleating protein. 40 h after transfection, cells were stimulated with arsenite, and the time-lapse fluorescence images were acquired every 5 min for 55 min. SGs were clearly observed at 10 min (green dots in Fig 1A). The size of SGs was small at this stage, but grew larger at a later time (50 min). In contrast, the number of SGs reached a maximum at 30 min, then decreased as a function of time. These dynamics to exemplify SG assembly are qualitatively shown in Fig 1B and 1C. A small number of SG assembly was observed at 5 min reaching a maximum (31.7 ± 2.7, N = 9) at 30 min, and then decreased to 22.0 ± 2.8 at 55 min (Fig 1B). During these observable changes in the number of SGs, the average size increased monotonically with time (Fig 1C). In this quantification, the size of SGs were measured by the number of pixels (**Materials and Methods**). S1 Movie demonstrates the overall dynamics clearly and was similar to previous reports [37,39–41]. To validate our SG size measurement, we quantified SGs differently by integrated fluorescent intensity aimed at SG size measurement by volume instead of diameter (**Materials and Methods**). The time course of SG assembly and its evolution of average size were not changed significantly (left and middle panels in S2A Fig).

### TIA-1 knockdown abrogated the SG assembly

**Fig 2:**
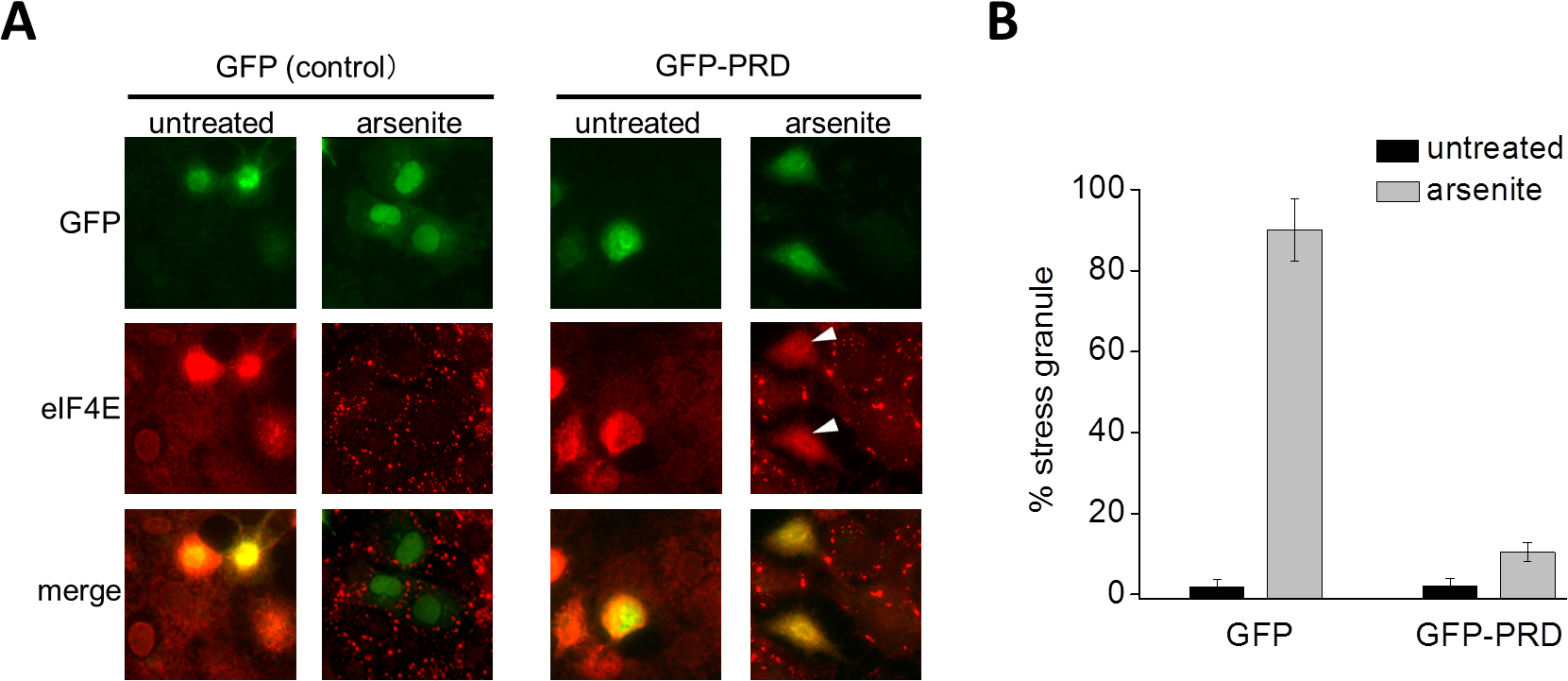
TIA-1 is required for the assembly of SG. (A) GFP alone or GFP-PRD was transiently transfected into COS-7 cells as indicated. After 48 h, the cells were treated with arsenite (0.5 mM) for 50 min. Endogenous eIF4E, a SG marker protein, was visualized by immunofluorescence. GFP-PRD-expressing cells treated with arsenite are indicated by arrow heads. (B) The percentage of GFP-positive cells containing SGs was determined and is shown in the graph. Error bars indicate s.e.m. (n = 3).

Next, the TIA-1 requirement for the assembly of SG was tested. TIA-1 possesses three RRMs at the N-terminus, along with a glutamine-rich PRD at the C-terminal region, both of which are essential for TIA-1-mediated SG assembly [9,29]. Previous studies reported that the expression of the C-terminal fragment of TIA-1, which contains the PRD alone, dominantly suppresses SG assembly [9,29]. Therefore, we constructed an expression vector encoding GFP-tagged PRD (GFP-PRD in S2B Fig), and examined if the expression of the PRD would affect the SG assembly. COS-7 cells were transiently transfected with either GFP alone (as control) or GFP-PRD, and incubated for 48 h. The cells were then treated with 0.5 mM arsenite for 50 min and the assembly of SGs was assessed by immunofluorescence microscopy (Fig 2A and 2B). In control cells expressing GFP alone, approximately 90 ± 76% of the cells formed SGs in response to arsenite. In contrast, cells expressing GFP-RPD scarcely showed SG assembly (10.4 ± 2.4%) (Fig 2B). These results clearly indicate that TIA-1 was required for the assembly of SG upon application of arsenite in COS-7 cells as was reported previously [8,29].

### Creation of a mathematical model for SG assembly

**Fig 3:**
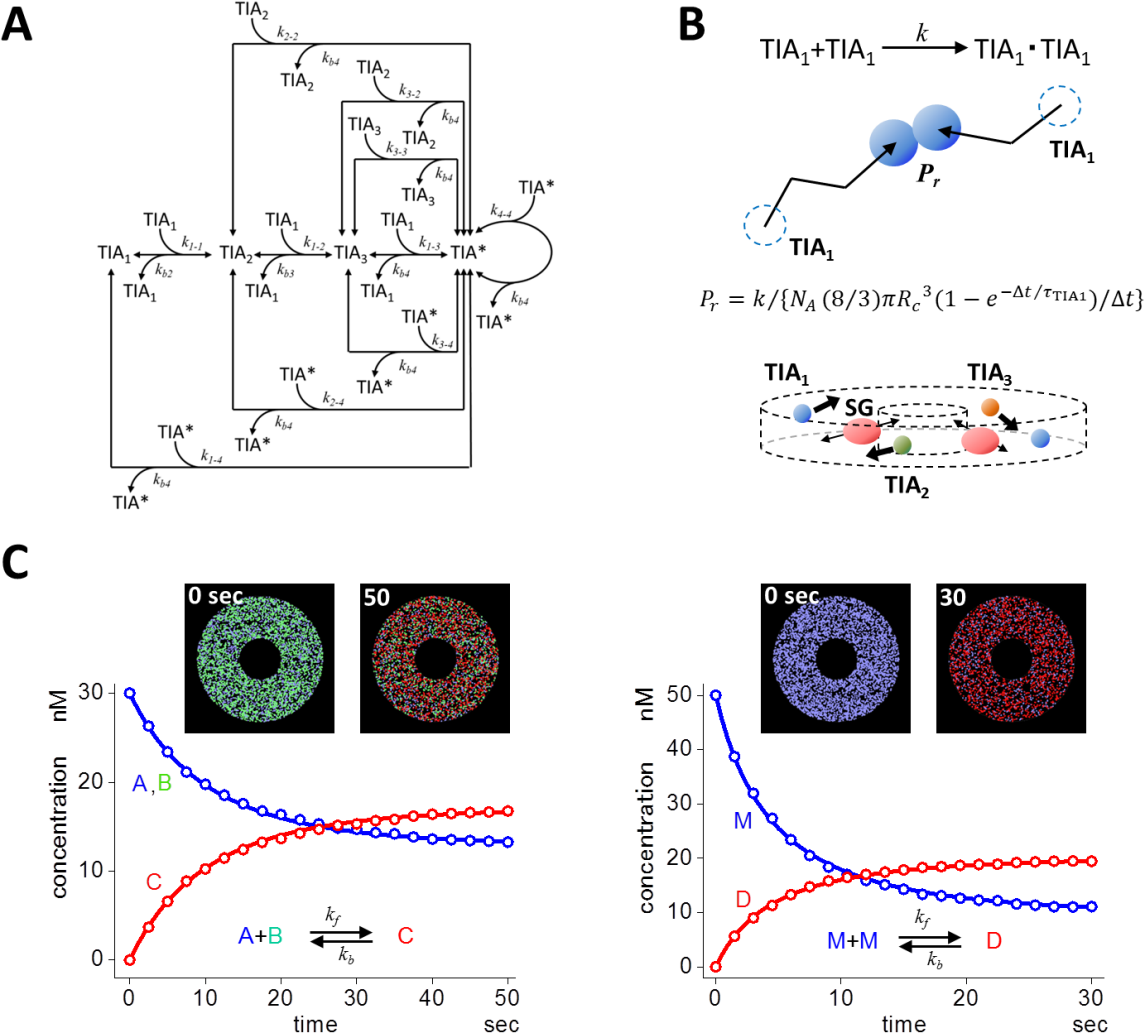
A model of SG assembly and its stochastic simulation. (A) A minimal model for SG assembly. Self-aggregation of TIA-1 upon application of a stress triggered the assembly of SGs. Single TIA-1 (TIA_1_) molecules formed dimer (TIA_2_), trimer (TIA_3_), and tetramer (TIA*). TIA* bound TIA_1_, TIA_2_, TIA_3_, and TIA* forming a larger TIA*. We assumed TIA* containing 12 or larger TIA_1_ as SG. (B) In our SS, molecules underwent RW jumping from the present position to the next (top panel). Two TIA_1_ molecules made a chance collision, and underwent dimer formation described by a probability *P*_*r*_, which was calculated according to an equation shown in the figure. All molecules underwent RW in a 3D circular model cell (bottom panel). SGs underwent 1D radial RW assuming transportation on microtubules, while other species underwent 3D RW. (C) SS was validated using simple reactions in a 3D circular model cell. SS results (red and blue open circles) agreed quite well with DS results (red and blue continuous curves) for the binding and dissociating bidirectional reaction (left panel). Initial conditions were as follows: [A] = [B] = 30 nM; [C] = 0 nM; *k*_*f*_ = 2x10^6^ /M/s; *k*_*b*_ = 0.02 /s. For a reaction of dimer formation, SS results also showed quite good agreement with DS (right panel). Initial conditions were as follows; [M] = 50 nM; [D] = 0 nM; *k*_*f*_ = 2x10^6^ /M/s; *k*_*b*_ = 0.01 /s. Insets in both panels are snapshots at the beginning and at the end of simulation. Each dot corresponds to each molecule, and the color correspond to molecular species shown in the reaction scheme in left and right panels.

We focused on the spatio-temporal dynamics of TIA-1-dependent SG assembly (i.e. time courses of the number and the size, and the spatial distribution) in a whole cell. We intended to construct a minimal model instead of a consolidative one that included all pathways shown in S1 Fig. Thus, in the model, SG assembly was initiated by and depended on the self-aggregation of TIA-1 (Fig 3A) because our experiments clearly showed the requirement of TIA-1 (Fig 2). Neither translocation of TIA-1 from the nucleus to the cytoplasm nor de novo synthesis of TIA-1 upon stress application was included in our model, because our experimental data excluded these possibilities (S3 Fig). In addition, TIA-1 translocation was not seen in data from other laboratories [44,45]. The O-GlcNAc modification of proteins in the translational machinery [46] was also excluded for simplicity.

The latency before the assembly of SGs was observed both in our (Fig 1B) and other laboratory’s experiments [39]. Latencies are often observed in biological phenomena. In signal transductions, latency emerges after multiple steps of activation cascade, which acts as rate limiting steps. Among them, the assembly of filamentous actin (F-actin) is one typical example [47–52]. The oligomerizations of globular actin (G-actin), which are nucleation steps, are the rate-limiting steps in F-actin assembly. In light of this, we employed a model in which a single TIA-1 (TIA_1_) formed a dimer (TIA_2_), and a trimer (TIA_3_) (3-step model shown in Fig 3A). These were the rate-limiting steps in SG assembly. Then TIA_3_ bound with TIA_1_ assembling TIA* containing four TIA_1_ molecules. TIA* further bound with TIA_1_, TIA_2_, TIA_3_, and TIA* resulting in the formation of a larger TIA*aggregate. These steps of SG enlargement are analogous to the elongation step of F-actin. SGs thus formed were assumed to be transported on microtubules. In the present model, TIA_1_ was a complex of TIA-1, mRNA and its binding proteins for simplicity.

In our SS, molecules undergo random walk (RW) by changing their location upon stochastic jumps in 3D space. During a jump, two TIA_1_ undergo a chance collision (top panel of Fig 3B). This can lead to a binding reaction between two TIA_1_s. The probability of the binding reaction *P*_*r*_ is calculated by Eq 1 (**Materials and Methods**), which is a function of the classical rate constant *k*, time between a jump τ, which is calculated from diffusion coefficient *D*, collision radius *R*_*c*_, and calculation time step *Δt*. The important aspect of *P*_*r*_ is that its usage guarantees the convergence of SS to DS at an infinite number of molecules for any selection of *τ*, *R*_*c*_, or *Δt* as long as *P*_*r*_ *<1* [43]. Our SS guarantees this, and we used the same theory and algorithm in the present SSs. The mathematical bases of our SS method are found elsewhere [43,53].

In the SS of SG assembly, TIA_1_, TIA_2_, and TIA_3_ underwent RW in a 3D model cell with a diameter and thickness of 12 and 1.5 μm, respectively (bottom panel of Fig 3B). A nucleus at the center with a diameter and thickness of 4 and 1.5 μm was located. SG movement along microtubules was reported [37,54]. We assumed that TIA* with 12 or larger number of TIA_1_s, which was tentatively defined as SG, underwent 1D radial RW simulating transportation on microtubules. Since hindered diffusion of large particles such as SGs was reported [2,55], SG transportation on microtubules was expected to lead to an effective collision between large SGs resulting in a replication of experimentally observed SG dynamics, which are discussed in the following sections.

Coordinates and state of every molecules including SG were stored in tables within the SS program, and were updated upon the occurrence of an event (jump, collision, and reaction). The number of TIA_1_ molecules in a single TIA* (and also SG) was stored in the table of the SS program. The size of SG was calculated using this table (**Materials and Methods**), and the total number of TIA_1_ in a 3D model cell, which is the summation of the number of TIA_1_s in all complexes including monomers, was monitored and kept constant.

To test the validity of our SS in the 3D model cell shown above, we ran simulations of simple reactions. SS results for the binding reaction (blue and red open circles in the left panel of Fig 3C) agreed almost perfectly with those of DS (blue and red lines). The initial number of molecules were 2724 for A and B, and 0 for C. SS results for dimerization reaction, which appeared in the present SG simulation, also agreed almost perfectly with those of DS (right panel in Fig 3C). Insets are snapshots at the beginning and at the end of SSs. These tests of SSs showed clearly that our SS could be applied to the simulation of SG assembly. All SS parameter values in the SG simulation are summarized in S1 Table. *P*_*r*_s for each reaction were calculated by the SS program using Eq 1 with given parameter values of *k*, *D*, *R*_*c*_, *τ*, and *Δt* before the start of SS (Cf. **Material and Methods**).

### SS of SG assembly replicated experimental observations

**Fig 4:**
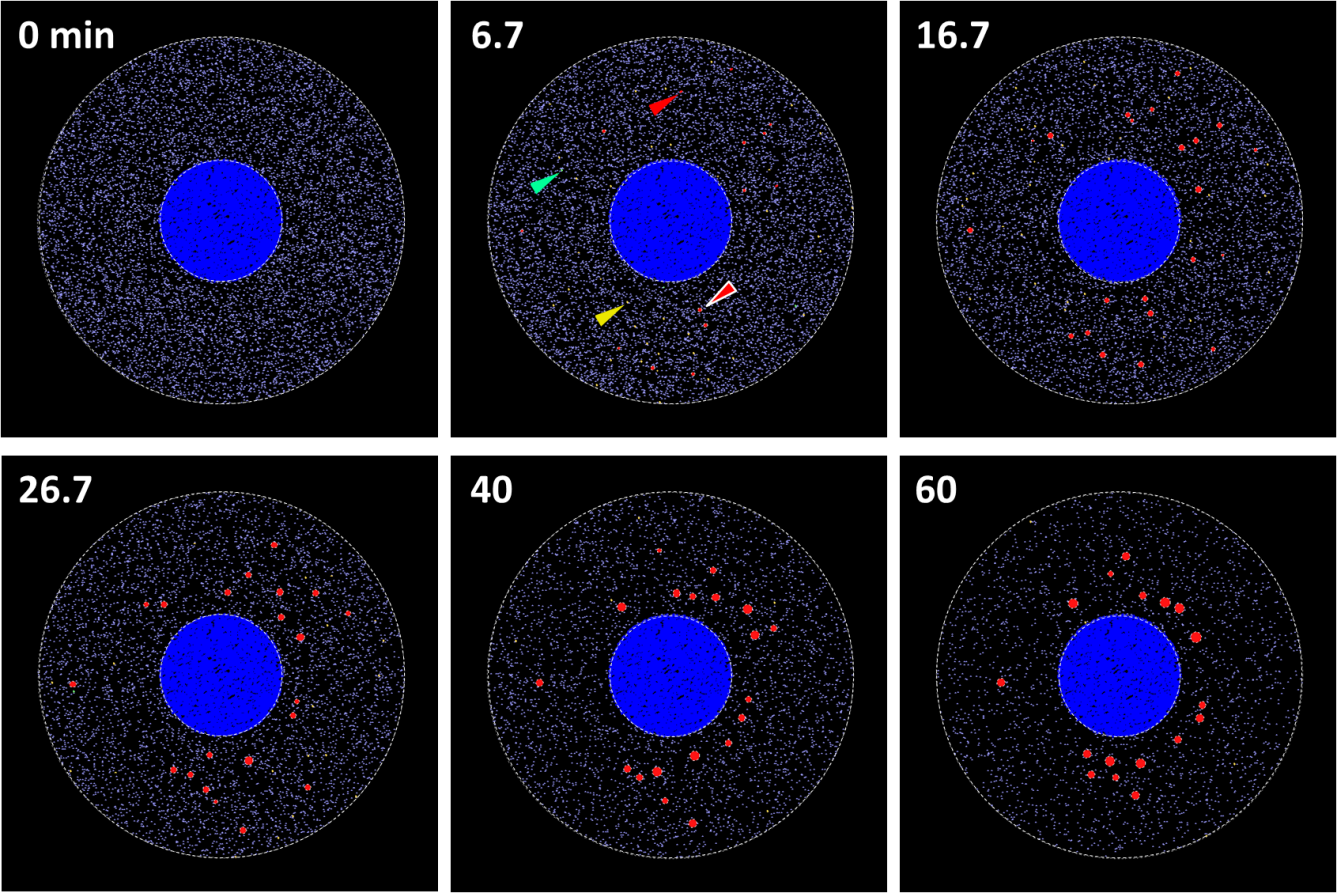
Simulation results of SG assembly. At 0 min, TIA_1_ (blue dots) distributed diffusely in the cytoplasm. At 6.7 min, several small SGs were assembled (red circles, one of which is indicated by a red arrowhead surrounded by white line). TIA_2_ (green dots, one of which is indicated by a green arrowhead), TIA_3_ (yellow dots, one on which is indicated by an yellow arrowhead), and TIA* (red dots, one of which is indicated by a red arrowhead) are also shown. At 16.7 min, SG distributed with no spatial preference. At 26.7 and 40 min, the size of SGs increased, and they gradually localized to the perinuclear region. At 60 min, the number of SG decreased, and the localization around nucleus became evident.

We ran SSs with an initial number of TIA_1_ equivalent to 9081 (blue dots at 0 min in Fig 4), which corresponded to a concentration of 100 nM in our 3D model cell. Each dot in Fig 4 indicates a single molecule with different colors for different molecular species. At 6.7 min, a small number of TIA_2_ (yellow dots, one of which is indicated by a yellow arrowhead) and TIA_3_ (green dots, one of which is indicated by a green arrowhead) were formed, and a small number of small SGs were also found (red circles, one of which is indicated by a red arrow surrounded by white line). TIA* containing TIA_1_ smaller than 12 are indicated by small red dots (one of which is indicated by a red arrowhead). At 16.7 min, both the number and the size of SGs increased. SGs gradually moved to perinuclear regions at later points in time (26.7, 40, and 60 min). These dynamics are clearly seen in the S2 Movie. Note that the number of TIA_1_ decreased with time as seen by the clearing up of blue dots from the background in the cytoplasmic space.

**Fig 5:**
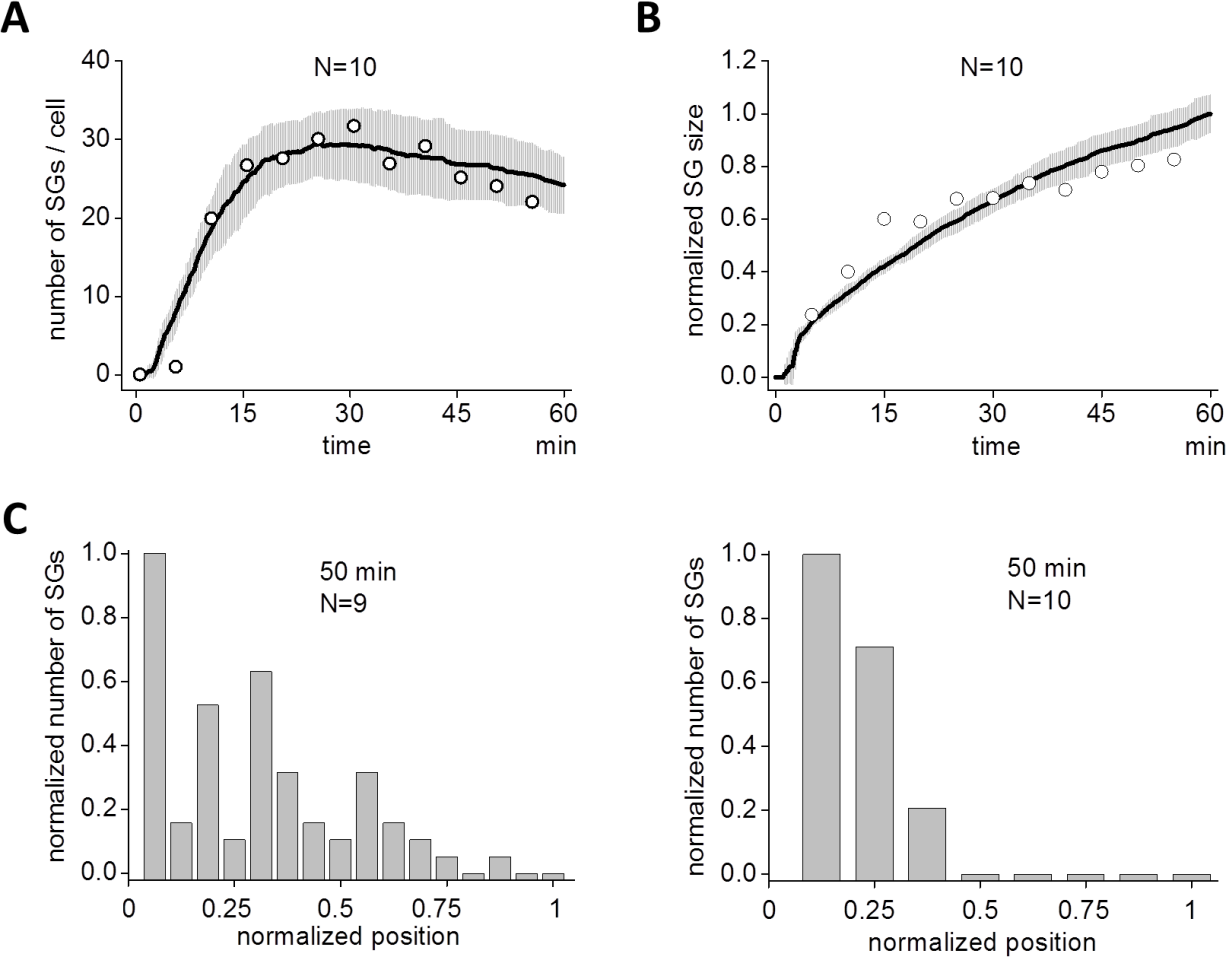
Spatio-temporal dynamics of simulated SG assembly. (A) The time course of assembled SG in SS also displayed a short latency followed by a peak and a decline (continuous line). Gray areas indicate SD by 10 SSs. Circles are replotted from experimental data in Fig 1B for comparison between SS and experiment. (B) Size of SG in SS (continuous line) developed sublinearly as was seen in the experiment. Circles are replotted from Fig 1C for comparison. (C) Experimental and simulated distributions of SG at 50 min are shown. SGs were localized at perinuclear region both in experiment (left) and SS (right).

If we plotted the number of SG as a function of time, SS results (black line in Fig 5A) agreed well with our observations (white circles, which were replotted from Fig 1B). Gray areas indicate standard deviation (SD) at each time point for multiple SSs (N = 10). SS with smaller runs (N = 5) gave almost the identical result (S4 Fig). This suggests that experimental data with N = 9 is sufficient for analyses. The size of SGs increased monotonically as discovered in the experiment (black line in Fig 5B). White circles were replotted from Fig 1C. Note that the time courses were sublinear both in the experiment and the SS.

Next we compared the simulated special distribution of SG with that of experiments. SGs were localized around the nucleus at 50 min during the experiments (left panel in Fig 5C). We found the same distribution in SSs at 50 min (right panel in Fig 5C). There were negligible SGs seen at a distance from the nucleus. If this was compared to an earlier time, distributions from both experiments (35 min) and simulations (35 min) agreed qualitatively (S5A and S5B Fig). Thus we replicated the dynamics of SG assembly qualitatively in our SSs.

We tested a simpler model than that shown in Fig 3A, where there were two molecular species, TIA_1_ and TIA* (1-step model shown in S6A Fig). TIA* that contained 12 or larger number of TIA_1_ was defined as SG as in the model shown in Fig 3A. SS results showed that neither latency nor the peak in the time course of the number of SG was found. If we plot the time to 99% of the saturated number of assembled SG by varying *k*_*f1*_ and *k*_*f2*_ in the model shown in S6A Fig, it was far shorter than the experimental observation on the time to peak (S6B Fig). Thus, this simple model could not replicate our experimental observations. We also tested a complex model by employing TIA_4_ in addition to TIA_1-3_ (4-step model shown in S6C Fig). This model also replicated the observed time course of number of SGs (S6D Fig).

Next we compared latencies from three SS models and our experiment. The latency was defined as the time to 10% of the maximum number of SGs, which was 5.6 min in our experiment (S7A Fig). Latencies for 1-, 3-, and 4-step models were 8.3x10^-3^, 3.8, and 4.2 min, respectively (S7B Fig). These results clearly showed the importance of multiple steps before the assembly of SG.

We also tested a DS model, which was based on ‘winners-share-all’ dynamics (S8A Fig). This mechanism was consistent with the observation that almost all SG resources (TIA-1) were collected and shared into a small number of SGs during the course of their assembly [11]. Snapshots of DS results seemed to be consistent with observations (upper panels of S8B Fig). However, there was no latency in the time course of the number of SG (lower panel of S8B Fig). Furthermore, this type of simulation could not involve SG transportation on microtubules, and therefore, neither movement on microtubules of assembled SGs nor their fusion could occur. In fact, large SGs, shown by reddish dots, did not move at all (S3 Movie), which was inconsistent with our experimental observation (Cf. S1 Movie). Thus a DS model shown here was not representative of experimental observations of SG assembly.

### Fusion and transportation of SGs were important for the dynamics of SG assembly

**Fig 6:**
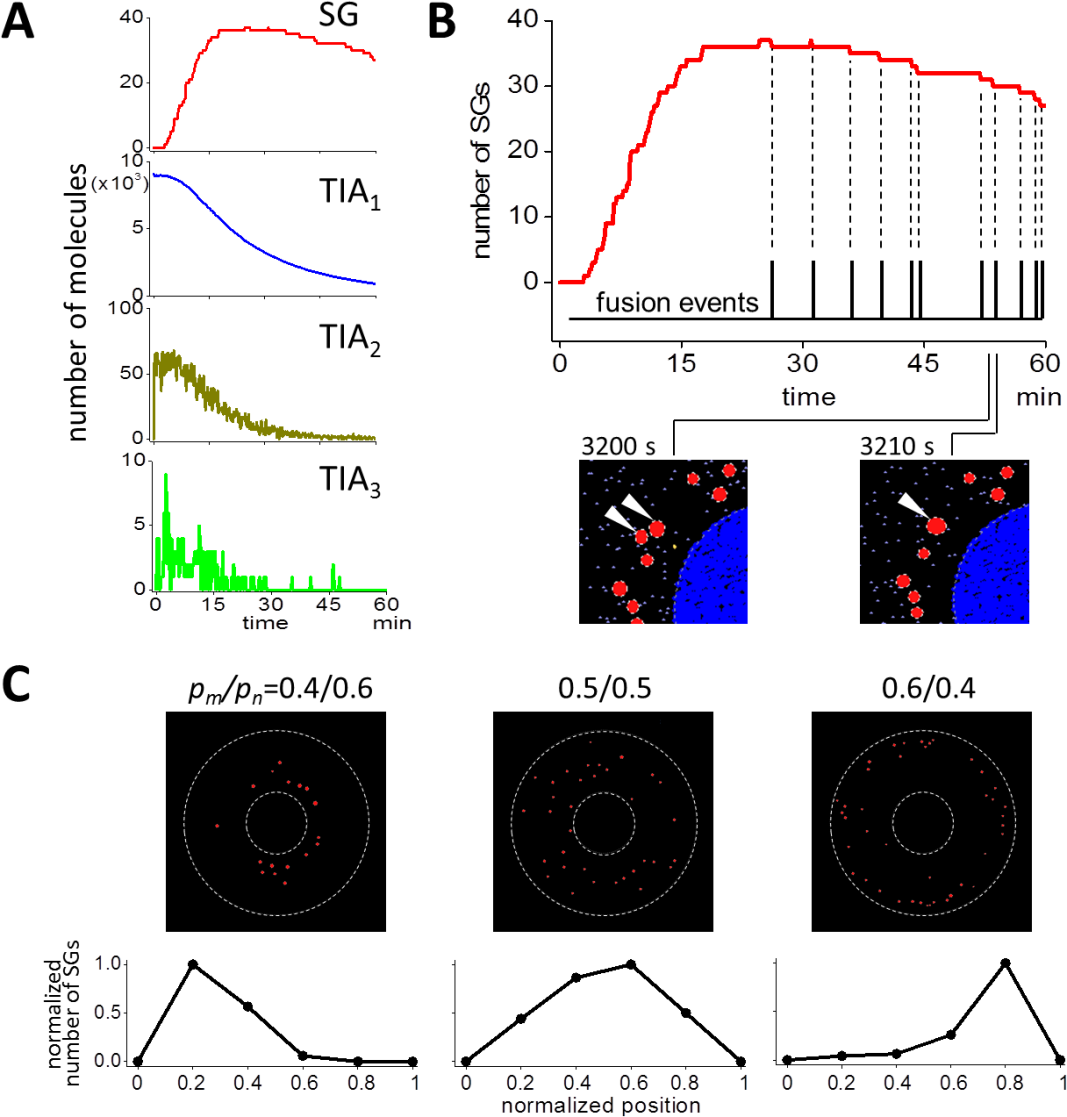
Analysis of the dynamics of SG assembly. (A) Time courses of SG, TIA_1_, TIA_2_, and TIA_3_ are shown. TIA_1_ decreased monotonically as the increase in the number of SG. TIA_2_ and TIA_3_ increased quickly just after the beginning of SS, and soon they decreased. The peak number of TIA_2_ was small, and that of TIA_3_ was 9, which was smaller than the peak number of SG. (B) The decrease in the number of SG after the peak was caused by the fusion between SGs. If we aligned the occurrences of fusions (black pulsatile vertical lines) to the time course of the number of SGs (red line), the occurrences of the decrement in the time course and those of fusion events coincided perfectly. Two SGs at 3200 sec (white arrowheads in the bottom left panel) fused together forming one larger SG at 3210 sec (white arrowhead in the bottom right panel). (C) SG distribution at 60 min was changed by the change in *p*_*m*_/*p*_*n*_. Small change in *p*_*m*_/*p*_*n*_ changed the SG distribution.

Next we investigated the dynamics of each molecular species in the model. First we analyzed the time course of complexes (Fig 6A). As the increase in the number of SG, TIA_1_ decreased monotonically. TIA_2_ and TIA_3_ increased rapidly just after the start of the simulation, and then decreased monotonically. The peak number of TIA_2_ and TIA_3_ was 66 and only 9, respectively in this SS. The small number of TIA_3_ was expected from Fig 4. This indicates that TIA_3_ lifetime was relatively short, and quickly made a transition back to TIA_2_ or forward to TIA*.

The number of SG after a peak decreased (Fig 5A). In this decreasing phase, stepwise decrements in the number of SG in a single SS were observed in SSs (top panel of Fig 6B). These decrements should be correlated to some event. We hypothesized that the fusion of SG occurred at time points of these decrements, and found that each decrement coincided exactly with the occurrence of single fusion events. In fact, two SGs at 3200 sec (white arrowheads in the bottom left panel in Fig 6B) fused into one larger SG at 3210 sec (white arrowhead in the bottom right panel). Thus, in our simulation, the fusion of SGs was a major reason for the decrease in the number of SGs after attaining its peak.

Next we tested the spatial distribution of SGs. In our simulations, the probability of antero- and retrograde transport were 0.4 (*p*_*m*_) and 0.6 (*p*_*n*_), respectively, resulting in perinuclear localization of SGs at 60 min (left panels of Fig 6C). It was expected that if these probabilities were changed, the distribution would also be changed. In fact, SG distributed dispersedly within the cytoplasm (middle panels) or near plasma membranes (right panels) for *p*_*m*_/*p*_*n*_ of 0.5/0.5 and 0.6/0.4, respectively. Thus the probability of antero- and retrograde transport of SGs on microtubules mainly determined the SG distribution in our simulation.

### Predicted SG size distribution and parameter sensitivity in the assembly of SGs

**Fig 7:**
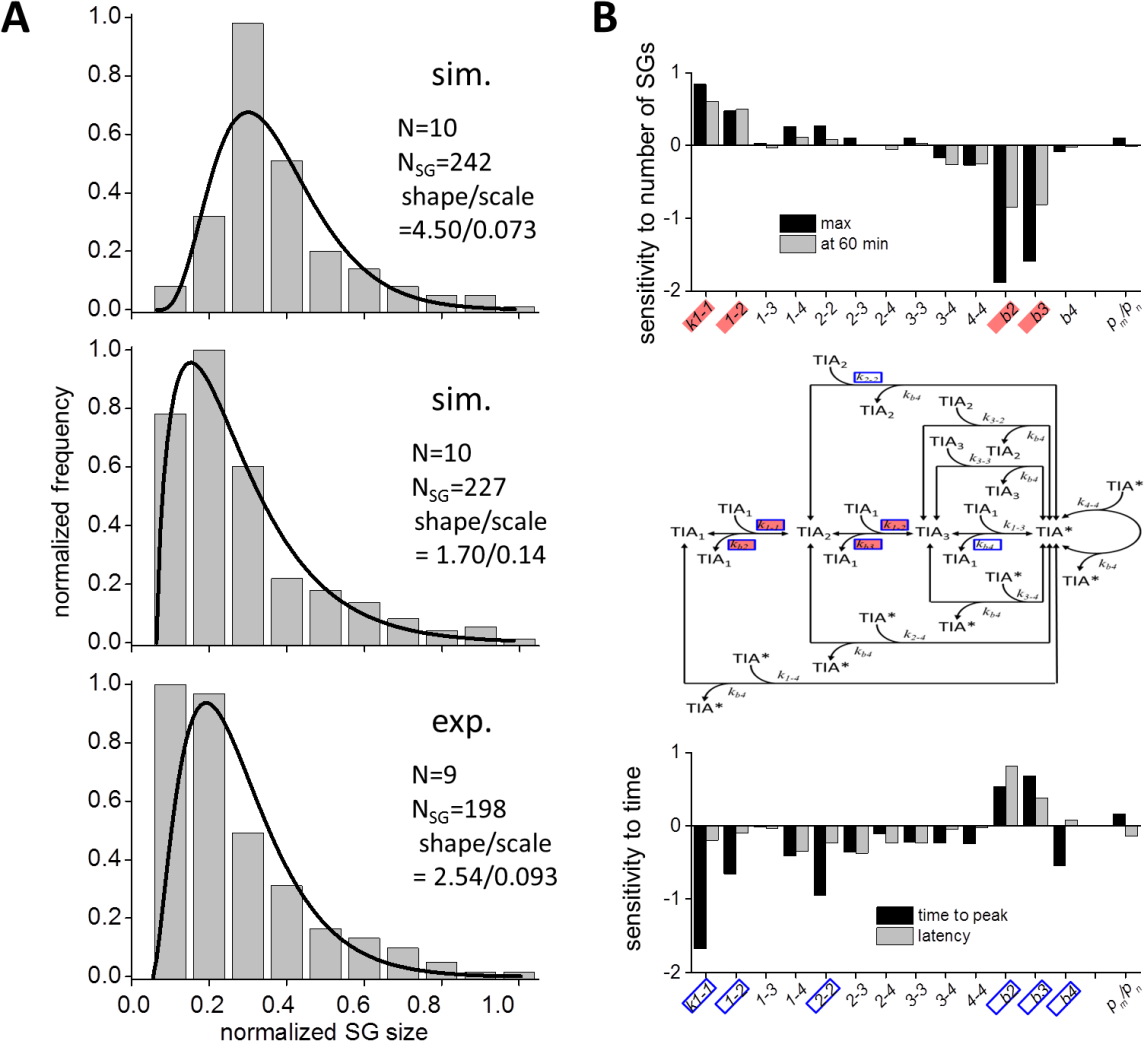
Gamma distribution of SG size both in SS and experiments, and the sensitivity of the dynamics of SG assembly to parameters. (A) The distribution of SG size at 55 min resembled gamma distribution both in SSs (top and middle panels) and experiments (bottom panel). Continuous lines are curves fitted by gamma distribution. The middle panel was drawn by eliminating SGs that contained smaller number of TIA-1 than 139 in the top panel mimicking limited detection of small SGs in our fluorescence microscopy. (B) Sensitivity of the dynamics of SG assembly. The numbers of SG at the peak (black bars) and at 60 min (gray bars) were sensitive to *k*_*1-1*_, *k*_*1-2*_, *k*_*b2*_, and *k*_*b3*_. They were virtually insensitive or only weakly sensitive to other parameters (top panel). The latency and the time to peak were sensitive to *k*_*1-1*_, *k*_*1-2*_, *k*_*2-2*_, *k*_*b2*_, *k*_*b3*_, and *k*_*b4*_. But they were almost insensitive or only weakly sensitive to other parameters (bottom panel). Rate constant affecting the SG number and time courses are shown in red and blue rectangles, respectively in the model of SG assembly (middle panel).

We were interested in the SG size distribution, because it might provide us with a better perspective on the dynamics of SG assembly. Bars in the top panel of Fig 7A indicate SG size distribution at 55 min as a result of the simulation, which was fitted by a gamma distribution with a shape and scale parameters of 4.50 and 0.073, respectively. The distribution in experiments at 55 min was also fitted by a gamma distribution with a shape and scale parameters of 2.54 and 0.093, respectively (lower panel of Fig 7A). There were significant differences between the simulation and the experiment. To explore the reason, we hypothesized that small SGs were not detected in our fluorescence measurement, and estimated that the number of GFP-TIA1 molecules in the smallest SG was 139 (**Materials and Methods**). If we eliminated SGs smaller than this in our simulated data, the distribution was much similar to that by the experiment with a shape and scale parameters of 1.70 and 0.14, respectively (middle panel of Fig 7A). In addition, the shape and scale parameters were almost unchanged by the change in N from 5 to 100 (S9 Fig), suggesting that the SG size distribution was fitted reasonably well with a gamma distribution in our simulation. These results suggest that small SGs were not detected in our fluorescence measurement.

To further validate our SG size measurement, we draw SG size distribution using data by integrated fluorescent intensity (**Materials and Methods**). The SG size was again approximated by a gamma distribution with a shape and scale parameters of 3.58 and 0.059, respectively (right panel in S2A Fig). The fact that the SG size was fitted by a gamma distribution both in our experiment and SS suggests that the SG assembly is an accumulation of successive random events, rather than ensembles of single random events.

Next we analyzed the change in the dynamics of SG assembly by investigating the change in kinetic parameters using data shown in S10 and S11 Figs. First we investigated the sensitivity of number of SGs at the time of the peak and at 60 min. The peak number of SGs was sensitive to *k*_*1-1*_, *k*_*1-2*_, *k*_*b2*_, and *k*_*b3*_, but insensitive or only weakly sensitive to other parameters (black bars in the top panel of Fig 7B). In contrast, the number at 60 min was only weakly sensitive to parameters tested (gray bars in the top panel of Fig 7B). The time to peak was sensitive to *k*_*1-1*_, *k*_*1-2*_, *k*_*2-2*_, *k*_*b2*_, *k*_*b3*_, and *k*_*b4*_. But the latency was only weakly sensitive to parameters tested (bottom panel of Fig 7B). Sensitive parameters for the number and the time are shown in solid red and blue boxes in the middle panel of Fig 7B, respectively. Overall, the dynamics of SG assembly was mainly sensitive to rate-limiting steps, and thus they determined the dynamics of SG assembly.

Next we investigated the sensitive parameters for determining sublinearity seen in the evolution of the size of SG (Fig 5B). We found that the sublinearity was sensitive to *k*_*1-4*_ and *p*_*m*_/*p*_*n*_ (S12 Fig). Larger *k*_*1-4*_ or *p*_*m*_/*p*_*n*_ ratio increased the sublinearity.

### The role of microtubules unifying the dynamics of SG assembly

We investigated how microtubules contributed to the multiple aspects of SG assembly. To this purpose we deleted microtubules from our SS by removing 1D radial RW during the SG assembly. Thus SGs underwent 3D RW as other species (TIA_1-3_, and TIA*). The *D* was the same as that for 1D radial RW of SGs. The simulated deletion of microtubules dramatically changed the dynamics. SGs were distributed throughout the cytoplasmic space (bottom panel of S13A Fig. The peak of the spatial distribution shifted from perinuclear to distant position (S13B Fig), which was similar to that in the presence of SG transportation on microtubules with *p*_*m*_/*p*_*n*_ of 0.5/0.5 (middle panel of Fig 6C). There was no decrease in the number of SGs in the time course of their assembly (S13C Fig). SG was small and distributed at bins of smaller sizes (S13A and S13D Fig). In addition, the normalized SG size distribution was largely different from that in the presence of microtubules (S13E Fig). While the distribution skewed positively in the presence of microtubules, it skewed negatively in their absence. These results strongly suggest that three experimental observations, the decrease in the number of SGs after the peak, the spatial distribution, and the size distribution were regulated by a single common mechanism of 1D radial RW of SGs on microtubules.

## Discussion

We intended to elucidate possible mechanisms for the dynamics of SG assembly in a whole cell both by experiments and mathematical models. To the best of our knowledge, this is the first occurrence to demonstrate SSs of SG assembly in a whole cell model. The SSs result replicated our experimental observations. In addition, the SSs result predicted a gamma distribution describing the SG size, which was also found in our experiments. One of important roles of computer simulations is to show a common mechanism for multiple phenomena. Our SSs replicated multiple aspects of our experimental observations including the decrease in the number of SGs after the peak, perinuclear spatial distribution, and the gamma distribution of SG size with a common mechanism of 1D RW of SG on microtubules.

We employed 1D RW of SG in the radial direction. The assumption of radial structure of microtubules does not hold at perinuclear region. However, overall orientation can be assumed to be radial [56,57]. Thus we employed this simple assumption in our minimal model.

Although the involvement of dynein in the retrograde transportation of SGs was suggested [41,42], there is no firm evidence for the involvement of kinesin in the anterograde transportation of SGs. However, we observed anterograde movement of SGs in our experiments (Cf. S1 Movie). In addition, mRNP was reported to move anterogradely on microtubules driven by kinesin motor protein [37]. Therefore, we hypothesized that SGs was transported anterogradely on microtubules in addition to retrograde movement. We have shown that the SG distribution was modified by probabilities of antero- and retrograde transportation (*p*_*m*_ and *p*_*n*_) of SG (Fig 6C). If motor proteins associated with a SG are modified posttranslationally by constituents of a SG in cell type- and stress-specific fashion, the probability of the movement direction would be modified too, and the SG distribution will be different by cell type- and stress-specific fashion. This might have a direct relationship to the biological roles of SG.

The distribution of SG size was fitted with a gamma distribution both in SSs and experiments (Fig 7A and S9 Fig). It is important to suggest that if the shape and/or scale parameter is different for different type of cells or stresses, existence of different kinetics and/or modifications in SG assembly mechanisms might exist. Thus analysis of SG size distribution will give us additional information for clarifying the mechanisms of SG assembly. A limitation to detect small SGs by fluorescence microscopy could result in a gamma distribution of SG size with different shape and scale parameters (top and middle panels of Fig 7A). However, different quantification method of SG size by integrated fluorescent intensity also gave a gamma distribution (right panel of S2A Fig), suggesting that SG size was intrinsically gamma distributed.

Sublinearity in the SG size evolution in experiments was more pronounced than in SSs. (Fig 5B). We have shown that the sublinearity was regulated by *p*_*m*_/*p*_*n*_ (S12 Fig). This suggests that the distribution of SG and the sublinearity in the SG size evolution might have a relation. This result was beyond our expectation. Further experiments and simulations are required to further reveal its mechanism and biological role.

The difference in the latency between 3- and 4-step models was small, and the latency in a 4-step model was still shorter than the experiment. This may suggest more number of rate-limiting steps are required for replicating experimental observations. However, there is an another possibility of the existence of preprocess before TIA_1_. In any case, the present study strongly suggested the existence of multiple rate-limiting steps before the assembly of SGs. In some of the simulation cases, the latency was so long that there was no observable decrease in the number of SG during 60 min of simulations, nor could the peak time be identified (Cf. S14 Fig). If we carefully looked at the evolution of SG size, we found that there was a catastrophic decrease in the SG size before SG assembly in many long latency cases (blue arrow in the bottom right panel of S15 Fig). This should be caused by a stochastic disassembly of SGs, and in extreme cases, no SG would be assembled. For example, such cases were observed for large values of *k*_*b2*_ and *k*_*b3*_, where latency and the time to peak were not measured because of no SG assembly (Cf. S11 Fig).

In the present study, we could not compare the size of SGs between experiments and simulations in an absolute fashion, because we could not know experimentally how many TIA-1 existed in a SG. If this could be measured, models and kinetics would be greatly improved enabling a better replication of the experiments and proposing additional predictions to describe the dynamics of SG assembly. It is also important to know the minimum size of observable SGs and the number of TIA-1 molecules within it.

## Materials and Methods

### Plasmids

Full-length TIA-1 or a C-terminal fragment of TIA-1 (TIA-1-PRD) was subcloned into pEGFP vector by PCR.

### Cell culture and transient transfection

HeLa and COS-7 cells were maintained in Dulbecco’s modified Eagle’s medium (DMEM) supplemented with 10% fetal bovine serum (FCS), L-glutamate, penicillin and streptomycin. For transient transfection assays, cells grown on a 35-mm-diameter glass bottom dish were transfected with the appropriate expression plasmids using Effectene transfection reagent (QIAGEN) according to the manufacturer’s protocol. 36 h after transfection, culture medium was exchanged with fresh DMEM/10%FCS without phenol red. The cells were incubated for another 4 h and then stimulated with arsenite (0.5 mM).

### Fluorescence microscopy

Fluorescence images of the living cells were captured using a TE-2000E inverted microscope (Nikon, Japan), equipped with a Planfluor 40× objective (numerical aperture, 0.6), a CoolSNAP HQ charge coupled device (CCD) camera (Photometrics), and a xenon lamp. For GFP imaging, a B-2A filter set [a dichroic mirror (500 nm, a long-pass), an excitation filter (450–490 nm), an emission filter (515 nm cut-on); Nikon, Japan] was used. Image binning was set to 2×2. Fluorescence images were acquired every 5min from the same field. Fluorescence microscopic images of fixed cells were captured using an inverted Olympus IX81 microscope equipped with a QImaging Retiga EXi digital camera (IEEE1394) and the Universal Imaging Metamorph software (Molecular Devices).

### Live cell image analysis of SG assembly

The assembly of SGs was determined from the fluorescence images using the Metamorph software package. First, the region of the entire cell area and the cytoplasmic area were manually located and the position of the region of the interest (ROI) was set in each fluorescence image in a time-series stack. Next, a median filter (25×25) was applied to the raw images and the resulting images were used as the background images. Images of SGs were generated by background image subtraction, by which the background images were appropriately subtracted pixel-by-pixel from the raw images. The number and the size of the SGs were quantified using transfluor assay module of the Metamorph software package and the pit-detection algorithm with the parameter describing approximate minimum width of 4 pixels (~1.3 μm) was applied to the SG images. We then measured the area size of individual SG, the number of SGs in the cytoplasmic region of the cell, and the x and y coordinates of the each SG dot relative to the nuclear centroid of the cell. In addition, we applied different quantification method, in which a fluorescence spot was surrounded by a circle (ROI) using Find Spots command of Metamorph, and ROI higher than the threshold level of fluorescent intensity was defined as SG. Then, the spatial integration of fluorescent intensity within a ROI was calculated aimed at measuring the volume of a SG instead of its diameter. The false-positive dots were carefully checked by looking and manually eliminated from the measurement.

### Immunofluorescence staining and microscopic observation of stress granules

Cells grown on glass coverslips were transfected with appropriate plasmids as indicated. Cellular SG assembly was then stimulated with 0.5 mM arsenite for 50 min. The cells were then fixed with 1% paraformaldehyde in PBS for 10 min. After washing with PBS, the cells were permeabilized with 0.1% Triton X-100 for 5 min, and incubated in the blocking solution BlockAce (Snow Brand Milk Products) for 1 h. Cells were then incubated with anti-eIF4E (Santa Cruz) antibody for 50 min in PBS containing 2% BSA, washed three times with PBS, and incubated with an Alexa Fluor 546-conjugated rabbit anti-mouse antibody for 30 min. The stained cells on coverslips were washed three times with PBS and were mounted in FluorSave Reagent (Calbiochem). SG assembly was assessed by determination of the number of cells expressing at least two SGs per cell.

### Computational model

We assumed that a SG began to assemble by aggregating TIA_1_ upon stress application. TIA_1_ was assumed to contain TIA-1, mRNA, and related proteins for simplification. TIA_1_ forms dimer (TIA_2_), trimer (TIA_3_), and larger complex (TIA*). TIA* grew further by binding TIA_1_, TIA_2_, TIA_3_, and TIA*. Reaction probability *P*_*r*_ in SSs was calculated by the following equation [43]:

$$P_{r}=k/\{N_{A}\left(4/3\right)\pi R_{c}{}^{3}\left(2-e^{-\Delta t/\tau_{1}}-e^{-\Delta t/\tau_{2}}\right)/\Delta t\}.$$ (1)

*k*, *N*_*A*_, *R*_*c*_, and *Δt* are binding reaction rate constant between molecule 1 and 2, Avogadro’s number, collision radius, and calculation time step, respectively. *τ*_*1*_ and *τ*_*2*_ are waiting times before the jump for molecule 1 and 2, which were calculated by using *τ =* λ^*2*^ */6/D*. *λ* and *D* were jump lengths and diffusion coefficient.

The convergence of our SS to DS is guaranteed as long as *P*_*r*_<1 as shown in the main text, and a theory and analyses for this convergence are found in our previous paper [43]. *R*_*c*_ was used for testing an occurrence of a collision, and *P*_*r*_ was used for decision of actual occurrence of a reaction by the collision. Thus, *R*_*c*_ was not the direct parameter for reactions. In this sense, *R*_*c*_ was not an important parameter in our SS. However, it should be noted that the spatial accuracy of the occurrence of a reaction worsens by the increase in *R*_*c*_. In contrast, small *R*_*c*_ gives a better spatial accuracy, but *P*_*r*_ would be larger than 1. This leads to a strategy for the selection of *R*_*c*_, in which small *R*_*c*_ is selected as long as *P*_*r*_<1. *R*_*c*_s between TIA_1_, TIA_2_, and TIA_3_ were 20 nm according to this consideration. The same *R*_*c*_ was employed for the reaction between TIA* and other species (TIA_1_, TIA_2_, and TIA_3_). *R*_*c*_ between two TIA*s was calculated by the summed radii of two TIA*s. Each radius of a TIA* was calculated by the following equation:

$$R_{TIA*}=R_{0}\cdot n_{TIA1}{}^{1/3},$$ (2)

where *R*_*0*_ is 10 nm and *n*_*TIA1*_ is the number of TIA_1_ in a single TIA*. The *P*_*r*_ between two TIA*s was assumed to be 1. TIA* contained 12 or a higher number of TIA_1_ moved on microtubule by 1D RW. Other species underwent diffusion in a cytoplasmic 3D space.

### Simulations

Simulations were run on computers consisting of an Intel Core i7-4770 processor (3.4GHz) with Windows 8 OS (64 bit). SS program was written in C language. Parallelization by Open MP or MPI was not applied in the present SSs. The computational time was about 24 h for a 60 min simulation of SG assembly with an initial number of about 15,000 molecules. SS program running on Windows PC can be found in “doi.org/10.6084/m9.figshare.1295250” on the web. DS model was constructed by A-Cell software [58,59]. The model description files can be downloaded from http://www.ims.u-tokyo.ac.jp/mathcancer/A-Cell/A-CellModels/index.html.

### Data analysis

SS yields a different result for every run because of the use of random variables in the simulations. So we averaged SS results from 5 or 10 runs and calculated SD using Origin Pro 8.5.0 J SR1 from Origin Lab Corporation. Fitting of SG size distribution with gamma distribution was performed by using R with fitdsitr() function of MASS package.

Sensitivity *S*_*e*_ was calculated using the following equation:

Se=∂PCPC∂PSPS, (3)

where *P*_*C*_ and *P*_*S*_ are characterizing and simulation parameters, respectively. Sensitivities of a single parameter were calculated at each value in the given range of a parameter shown in S10 and S11 Figs, and they were averaged giving an average sensitivity in the range, which was used in Fig 7B. We defined a characterizing parameter as sensitive, when *S*_*e*_ was larger than 25% of the absolute maximum value of sensitivity for the number or the time in SG assembly.

We estimated the minimum size of a SG to be detected in our fluorescence measurements. It was reported that ~200 nM EGFP fluorescence signal could be detected above typical cellular autofluorescence [60]. We employed a 1.3 μm square window for detecting SGs as shown in the previous section. If spherical shape of a SG was assumed within the window, the number of GFP-TIA1 molecules in the smallest SG was estimated using the following equation:

$$n_{TIA1}=C_{f}\cdot \frac{4}{3}\pi r^{3}\cdot N_{A},$$ (4)

where *C*_*f*_, *r*, and *N*_*A*_ are 200 nM, 0.65 μm, and Avogadro’s number, respectively. These yielded *n*_*TIA1*_ of 139.

## Supporting Information

S1 Movie SG assembly by arsenite application in experiment. (MP4)

S2 Movie SG assembly by SS. (MP4)

S3 Movie SG assembly by DS. (MP4)

S1 Table Parameter values for 3-step SSs of SG assembly. (PDF)

S2 Table Parameter values for 4-step SSs of SG assembly. (PDF)

S1 Fig Translation initiation in eukaryotic cell and SG assembly by abrogated initiation. Translation initiation steps are shown in thin lines and narrow characters. It begins with the formation of eIF5 ∙ eIF2_*αβγ*_ ∙ GTP complex and binding of tRNA^Met^ to it. Stalled translation initiation and SG assembly process are shown in thick lines and bold characters. By the application of a stress (red lightning), α subunit of eIF2 is phosphorylated inhibiting the formation of GTP-bound eIF5 ∙ eIF2_*αβγ*_. This process is the prerequisite for the SG assembly. Many kinases are known in the phosphorylation of eIF2_α_. Their activation is specific to stress. TIA-1, which is a shuttling protein between the nucleus and the cytoplasm, possesses one prion related domain (PRD), through which it undergoes self-aggregation upon stress application. TIA-1 binds mRNP through RNA recognition motifs (RRMs). Thus stress application leads to the assembly of SGs. The involvement of GCN2, mTOR, G3BP, and polysome disassembly are also reported to be involved in the SG assembly. When assembled, SGs can fuse together, which leads to an assembly of a larger SG. Subscript ‘n’ and ‘c’ indicate a nuclear and cytoplasmic protein, respectively.
(TIF)

S2 Fig SG dynamics measured by integrated fluorescence intensity and schematic structure of the TIA-1 protein. (A) SG measurements by integrated fluorescent intensity (red circles) gave almost identical results to those by the number of pixels (black circles) in the time course of the number of SGs and their size (left and middle panels). SG size was also approximated by a gamma distribution if we measured the size of SGs by integrating fluorescent light intensity (red bars in right panel) instead of the number of pixels (black bars). (B) Each number indicates RNA recognition motif (RRM). PRD, prion-related domain. GFP-PRD is a chimeric protein, in which the PRD of TIA1 was fused to the C-terminus of GFP.
(TIF)

S3 Fig Whole cell GFP-TIA1 level and both whole cell and nuclear fluorescent intensities were not altered before and after the addition of arsenite. (A) The level of GFP-tagged TIA-1 was not changed by the addition of arsenite (top). Arsenite-induced eIF2α phosphorylation was confirmed by immunoblotting (middle). The expression level of total eIF2α is also shown (bottom). (B) The fluorescent intensities in the nucleus and the whole cell were not changed by the addition of arsenite during 55 min.
(TIF)

S4 Fig There was a small change in the time course of SG assembly if number of SSs was reduced to 5. The difference by the number of SS between 10 (red line) and 5 (black line) was small.
(TIF)

S5 Fig The distribution of SGs around the peak time of a number SG. The distribution at 35 min in experiment (A) and in simulation (B) shifted slightly right from those at 50 min.
(TIF)

S6 Fig Simpler and more complex SS models and their simulation results. (A) We tested a much simpler model for the assembly of SG, where no rate-limiting step was employed. (B) Simulation results of the simpler SS model with the canonical parameters as follows: *k*_*f1*_ = 2x10^5^ /M/s; *k*_*b1*_ = 0 /s; *k*_*f2*_ = 10^6^ /M/s; *k*_*b2*_ = 0.1 /s; *k*_*f3*_ = 2x10^6^ /M/s; *k*_*b3*_ = 0.1 /s. There was no latency, peak, and decay in the time course of SG assembly (left panel). If we measured the time to 99% of the plateau level in the number of SG (blue plane in the right panel), it was much shorter than the observed time to peak (30 min in our experiments shown as an orange plane in the right panel). Thus the simpler SS model did not agree with our experimental results. (C) A complex model with 4 steps before the assembly of TIA*. (D) Simulation results for the 4-step model (black line with gray area for SD) with parameters shown in S2 Table also agreed well with our experimental observation (open circles).
(TIF)

S7 Fig Comparison of latencies among experiment and different SS models. (A) Measurements of latencies from experimental and simulation results of different 3 SS models. (B) Latency for 1-step model was significantly smaller than that of experiment. Latencies for 3- and 4-step models were close to experiment.
(TIF)

S8 Fig DS model and the simulation results. (A) We also tested DS model employing winners-share-all mechanism. Stress stimuli with random interval caused the formation of S in the DS model. S activated TIA, and activated TIA (TIA*) was further activated into TIA**, which is a positive feedback process. mRNA (mR) is assembled into SG by TIA**. S, TIA** and TIA** underwent self-inactivation. *k*_*3_*_, which is a rate constant for TIA** inactivation, was assumed to be dependent on its concentration, which we assumed a measure of the size of a SG. These reaction schemes were embedded into 3D circular cell model (20 μm in diameter with a nucleus of 8.3 μm in diameter). (B) Simulation results of DS model were quantitatively agreed with our experiments (upper panels). However, there was no latency in the time course of SG assembly (lower panel). In addition, SGs could not be transported on microtubules, which did not agree with experimental observations.
(TIF)

S9 Fig Shape and scale parameters in gamma distribution were not changed by the change in the number of simulations. (A) We compared shape and scale parameters in different number of simulations of 5, 10, 20, 50, and 100. (B) The shape and scale parameters were almost unchanged. In addition, there was a small decrease in the variance, which was reasonable.
(TIF)

S10 Fig Parameter sensitivity in the number of assembled SG. Forward and backward rate constants in the model of SG assembly were changed to see the change in the number of SG at the peak (red lines) and at 3600 sec (black lines). Red and black broken lines indicate data in the experiments for comparison. The changes in the SG number by the change in *p*_*m*_/*p*_*n*_ are also shown. Canonical parameter values were as follows: *k*_*1-1*_ = 10^5^; *k*_*1-2*_ = 5x10^4^; *k*_*1-3*_ = 10^5^; *k*_*1-4*_ = 8x10^5^; *k*_*2-2*_ = 10^6^; *k*_*2-3*_ = 10^6^; *k*_*2-4*_ = 10^6^; *k*_*3-3*_ = 10^6^; *k*_*3-4*_ = 10^6^; *k*_*4-4*_ = 10^6^; *k*_*b2*_ = 1; *k*_*b3*_ = 0.1; *k*_*b4*_ = 3x10^-3^; *p*_*m*_ = 0.4; *p*_*n*_ = 0.6 (Units for forward and backward rate constants were /M/s and /s, respectively). One parameter was changed for the range shown in each graph leaving other parameters unchanged from canonical values. Missing data points, which are seen in the peak value at *k*_*1-1*_ lower than 10^5^ /M/s for example, indicate that there was no peak because of a big latency or no SG was assembled (Cf. S14 Fig). It is interesting to see that the difference between the peak value and that at 60 min increased as the increase in *k*_*1-1*_ and *k*_*1-2*_. See **Materials and Methods**for the calculation of sensitivity.
(TIF)

S11 Fig Parameter sensitivity in the latency and the time to peak. The sensitivity of the latency and the time to peak on kinetic parameters were tested as in S10 Fig. Forward and backward rate constants in the model for SG assembly were changed to see the change in the latency (black lines) and the time to peak (red lines). It is interesting to find that the latency was only sensitive to the smaller *k*_*1-1*_, *k*_*1-2*_, and *k*_*2-2*_. Red and black broken lines show data from our experiments. Methods of calculating sensitivity was the same as in S10 Fig.
(TIF)

S12 Fig The change in the subliniearity of the evolution of SG size. *k*_*1-4*_ and *p*_*m*_/*p*_*n*_ ratio changed the sublinearity.
(TIF)

S13 Fig SS in the absence of microtubules altered the dynamics of SG assembly considerably. (A) SS results with (top panel) and without (bottom panel) microtubules at 60 min. In the absence of microtubules, small SGs were distributed throughout the cytoplasmic space. (B) Spatial distribution of SGs in the absence of microtubules (noMT, red line) was largely different from that in the presence of microtubules with *p*_*m*_/*p*_*n*_ of 0.4/0.6 (MT, black line). (C) There was no decrease in the number of SGs during SG assembly in the absence of microtubules. (D) SG size distributed at smaller SG bins in the absence of microtubules (red bars). The histogram was normalized to the maximum frequency in the absence of microtubules. (E) While the SG size distribution skewed positively in the presence of microtubules (black bars), it skewed negatively in their absence (red bars).
(TIF)

S14 Fig Simulation result example showing no decrease in the number of SG. In case of long latency before the assembly of SGs, we could not observe a decrease in the number of SG during 60 min, and it was impossible to define the time to peak.
(TIF)

S15 Fig Catastrophic disassembly of SGs. In many cases of long latency before SG assembly (right panels), we saw catastrophic disassembly of SGs (blue arrow). In contrast, there was no such catastrophic disassembly in a simulation with normal latency (left panels).
(TIF)
