# Supplementary material for: Spatio-temporal Dynamics and Mechanisms of Stress Granule Assembly
Source: PLoS Comput Biol. 2015 Jun 26;11(6):e1004326. doi: 10.1371/journal.pcbi.1004326 (PMC4482703; doi:10.1371/journal.pcbi.1004326)
Supplement: S1 Table — (PDF) [file pcbi.1004326.s004.pdf]

Table S1 Parameters used for 3-step SS of SG assembly

Parameter	Value	Unit
TIA_1	9081	molecules
TIA_2	0	molecules
TIA_3	0	molecules
TIA*	0	molecules
k_{1-1}	$7x10^4$	/M/s
k_{1-2}	$5x10^4$	/M/s
k_{1-3}	10^5	/M/s
k_{1-4}	$2x10^6$	/M/s
k_{2-2}	10^6	/M/s
k_{2-3}	10^6	/M/s
k_{2-4}	10^6	/M/s
k_{3-3}	10^6	/M/s
k_{3-4}	10^6	/M/s
k_{4-4}	10^6	/M/s
k_{b2}	1	/s
k_{b3}	0.1	/s
k_{b4}	$3x10^{-3}$	/s
D_{TIA1-3}	10^{-11}	m^2/s
D_{TIA*}	10^{-16}	m^2/s
r_{cell}	6	μm
h_{cell}	1.5	μm
$r_{nucleus}$	2	μm
$h_{nucleus}$	1.5	μm
p_n	0.6	–
p_m	0.4	–
