# Supplementary figures and images for: Spatio-temporal Dynamics and Mechanisms of Stress Granule Assembly
Source: PLoS Comput Biol. 2015 Jun 26;11(6):e1004326. doi: 10.1371/journal.pcbi.1004326 (PMC4482703; doi:10.1371/journal.pcbi.1004326)

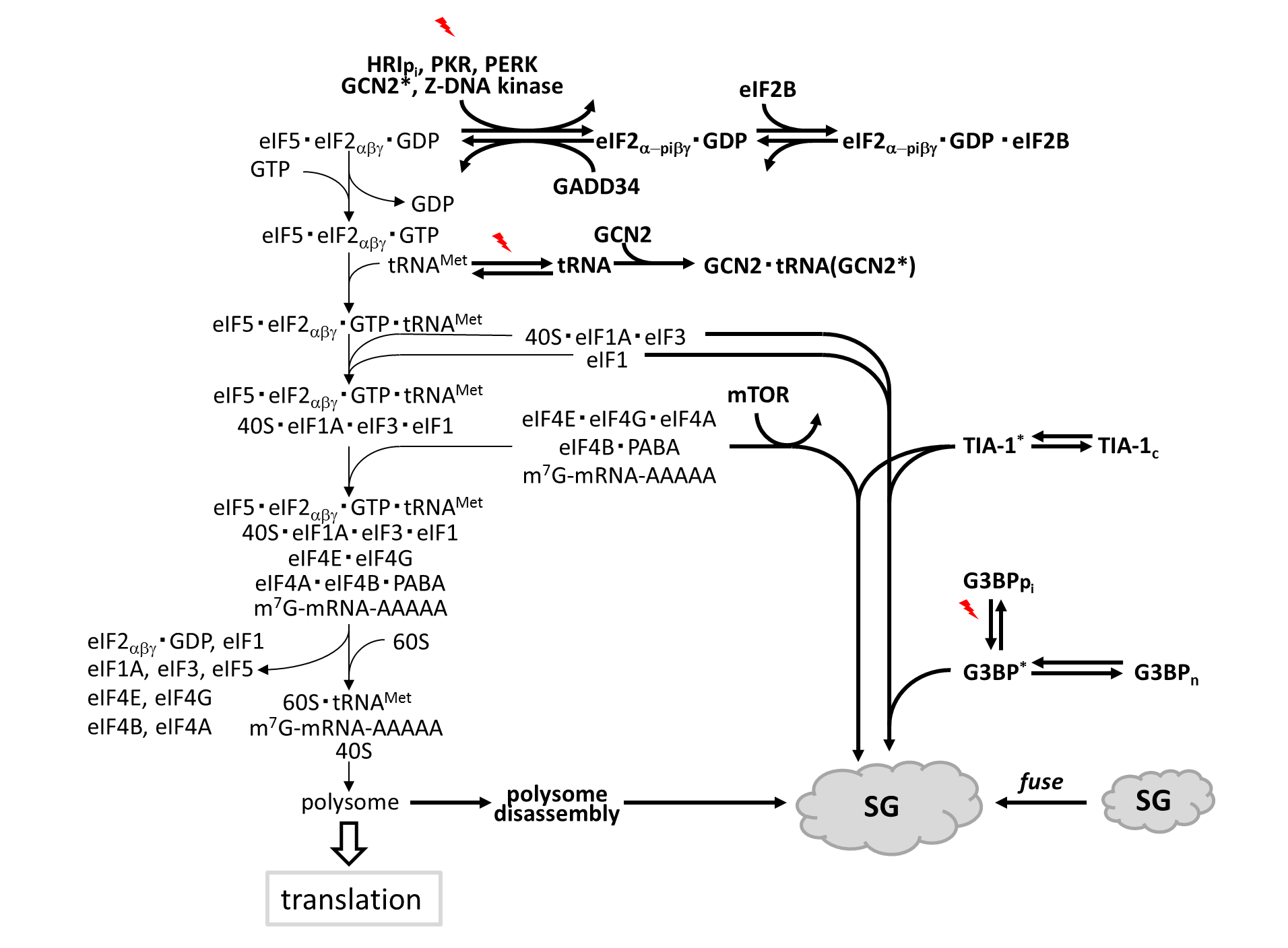

Supplement: S1 Fig — Translation initiation steps are shown in thin lines and narrow characters. It begins with the formation of eIF5 ∙ eIF2αβγ ∙ GTP complex and binding of tRNAMet to it. Stalled translation initiation and SG assembly process are shown in thick lines and bold characters. By the application of a stress (red lightning), α subunit of eIF2 is phosphorylated inhibiting the formation of GTP-bound eIF5 ∙ eIF2αβγ. This process is the prerequisite for the SG assembly. Many kinases are known in the phosphorylation of eIF2α. Their activation is specific to stress. TIA-1, which is a shuttling protein between the nucleus and the cytoplasm, possesses one prion related domain (PRD), through which it undergoes self-aggregation upon stress application. TIA-1 binds mRNP through RNA recognition motifs (RRMs). Thus stress application leads to the assembly of SGs. The involvement of GCN2, mTOR, G3BP, and polysome disassembly are also reported to be involved in the SG assembly. When assembled, SGs can fuse together, which leads to an assembly of a larger SG. Subscript ‘n’ and ‘c’ indicate a nuclear and cytoplasmic protein, respectively. (TIF) [file pcbi.1004326.s006.tif]

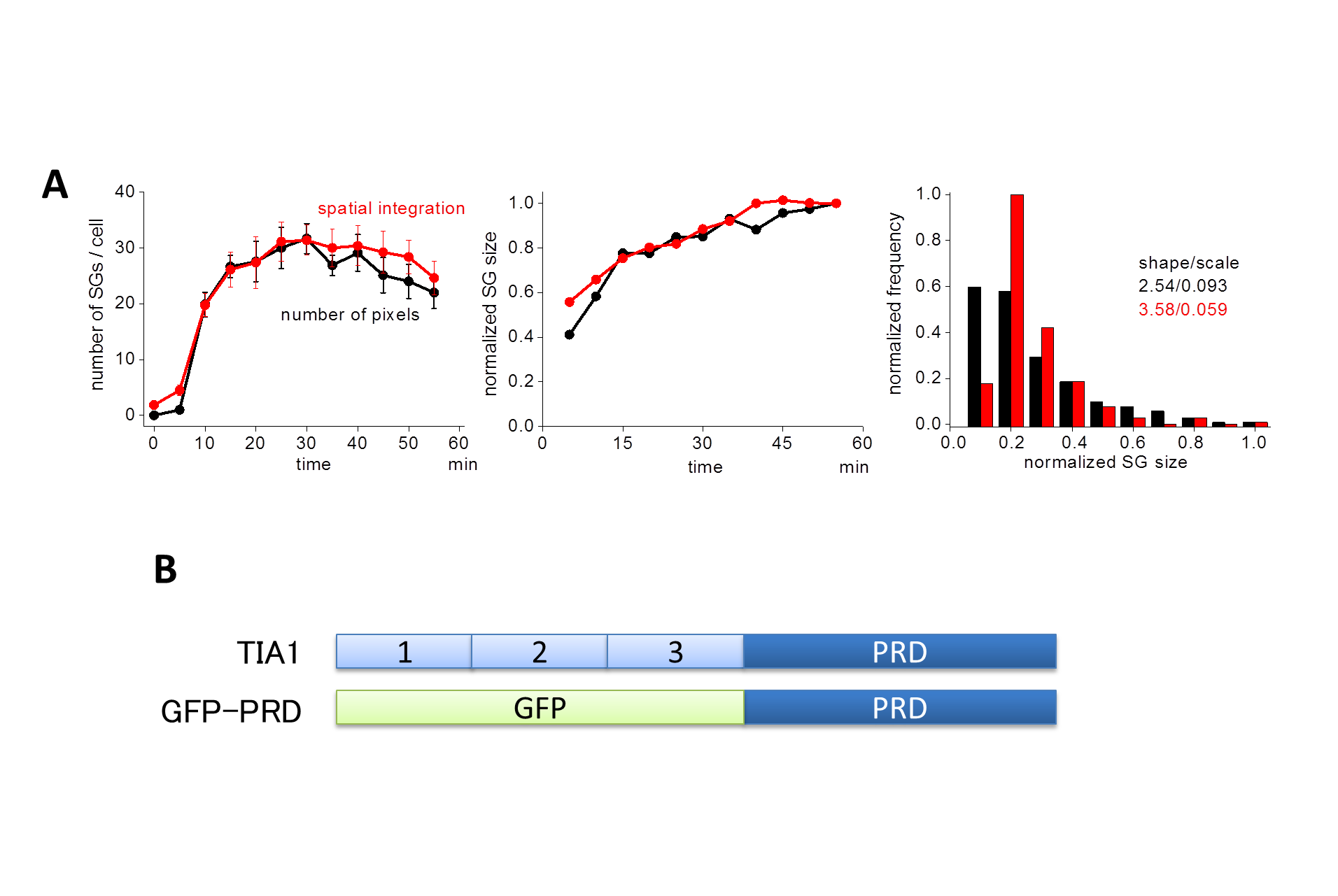

Supplement: S2 Fig — (A) SG measurements by integrated fluorescent intensity (red circles) gave almost identical results to those by the number of pixels (black circles) in the time course of the number of SGs and their size (left and middle panels). SG size was also approximated by a gamma distribution if we measured the size of SGs by integrating fluorescent light intensity (red bars in right panel) instead of the number of pixels (black bars). (B) Each number indicates RNA recognition motif (RRM). PRD, prion-related domain. GFP-PRD is a chimeric protein, in which the PRD of TIA1 was fused to the C-terminus of GFP. (TIF) [file pcbi.1004326.s007.tif]

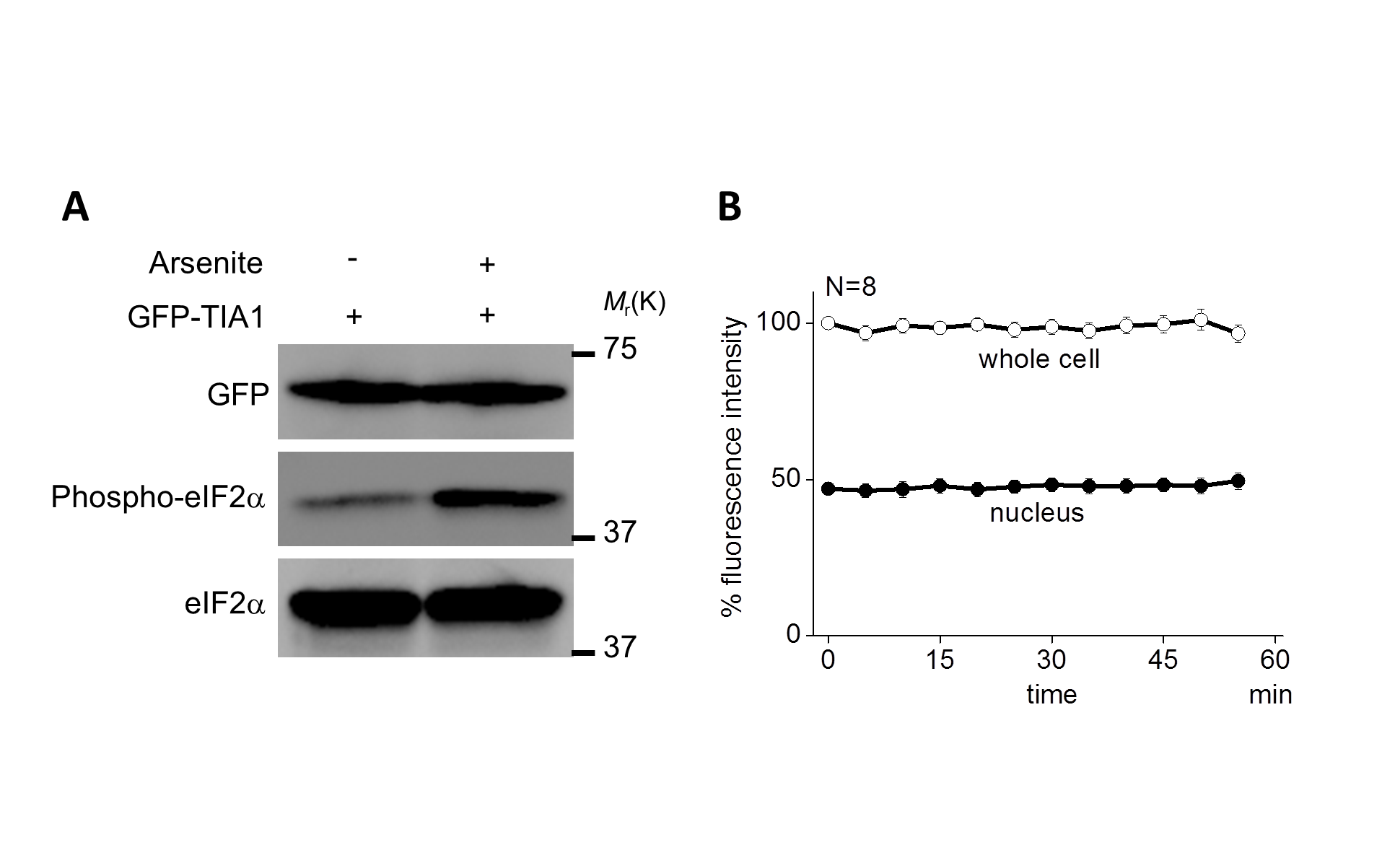

Supplement: S3 Fig — (A) The level of GFP-tagged TIA-1 was not changed by the addition of arsenite (top). Arsenite-induced eIF2α phosphorylation was confirmed by immunoblotting (middle). The expression level of total eIF2α is also shown (bottom). (B) The fluorescent intensities in the nucleus and the whole cell were not changed by the addition of arsenite during 55 min. (TIF) [file pcbi.1004326.s008.tif]

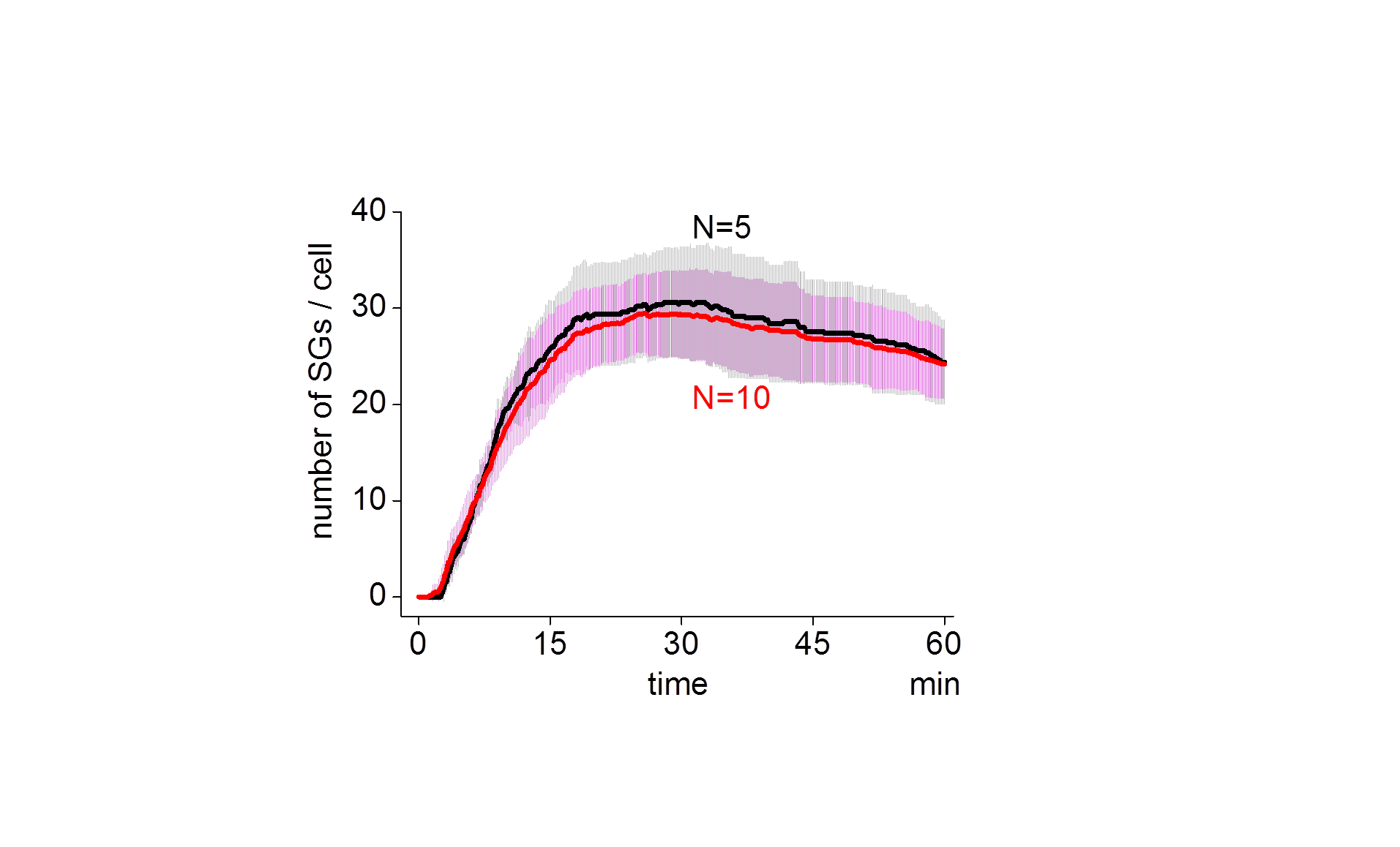

Supplement: S4 Fig — The difference by the number of SS between 10 (red line) and 5 (black line) was small. (TIF) [file pcbi.1004326.s009.tif]

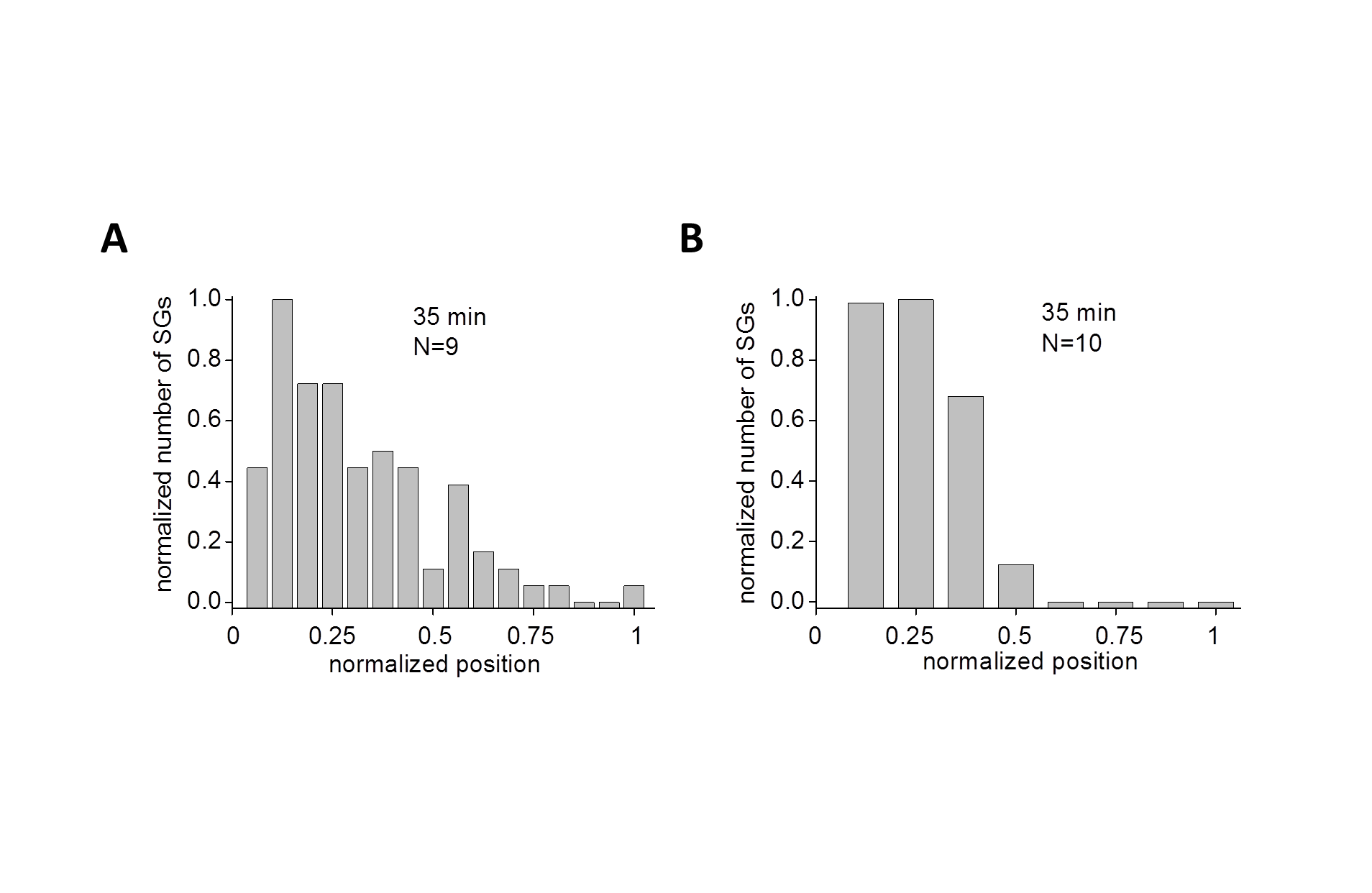

Supplement: S5 Fig — The distribution at 35 min in experiment (A) and in simulation (B) shifted slightly right from those at 50 min. (TIF) [file pcbi.1004326.s010.tif]

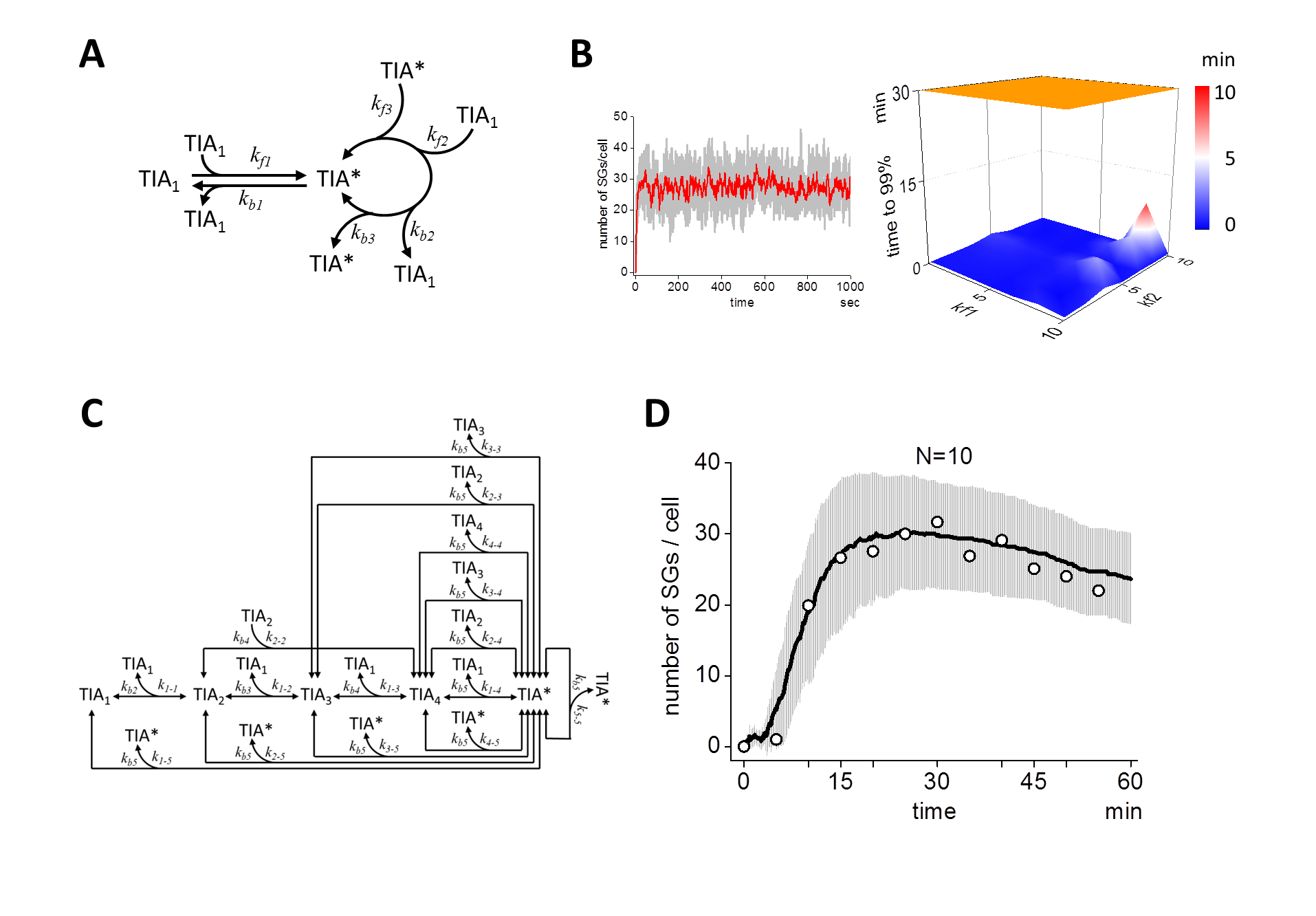

Supplement: S6 Fig — (A) We tested a much simpler model for the assembly of SG, where no rate-limiting step was employed. (B) Simulation results of the simpler SS model with the canonical parameters as follows: k f1 = 2x10^5^ /M/s; k b1 = 0 /s; k f2 = 106 /M/s; k b2 = 0.1 /s; k f3 = 2x10^6^ /M/s; k b3 = 0.1 /s. There was no latency, peak, and decay in the time course of SG assembly (left panel). If we measured the time to 99% of the plateau level in the number of SG (blue plane in the right panel), it was much shorter than the observed time to peak (30 min in our experiments shown as an orange plane in the right panel). Thus the simpler SS model did not agree with our experimental results. (C) A complex model with 4 steps before the assembly of TIA*. (D) Simulation results for the 4-step model (black line with gray area for SD) with parameters shown in S2 Table also agreed well with our experimental observation (open circles). (TIF) [file pcbi.1004326.s011.tif]

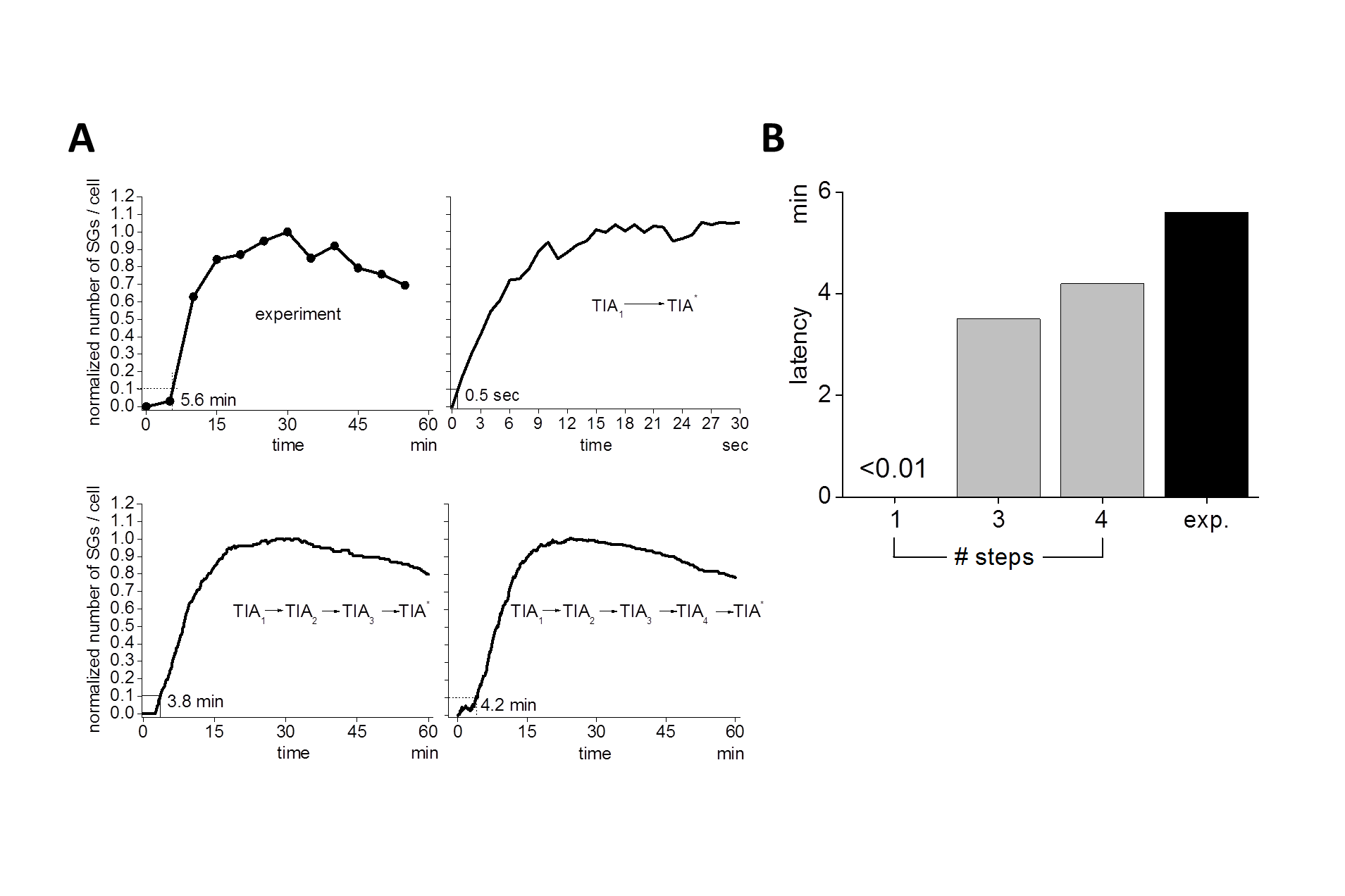

Supplement: S7 Fig — (A) Measurements of latencies from experimental and simulation results of different 3 SS models. (B) Latency for 1-step model was significantly smaller than that of experiment. Latencies for 3- and 4-step models were close to experiment. (TIF) [file pcbi.1004326.s012.tif]

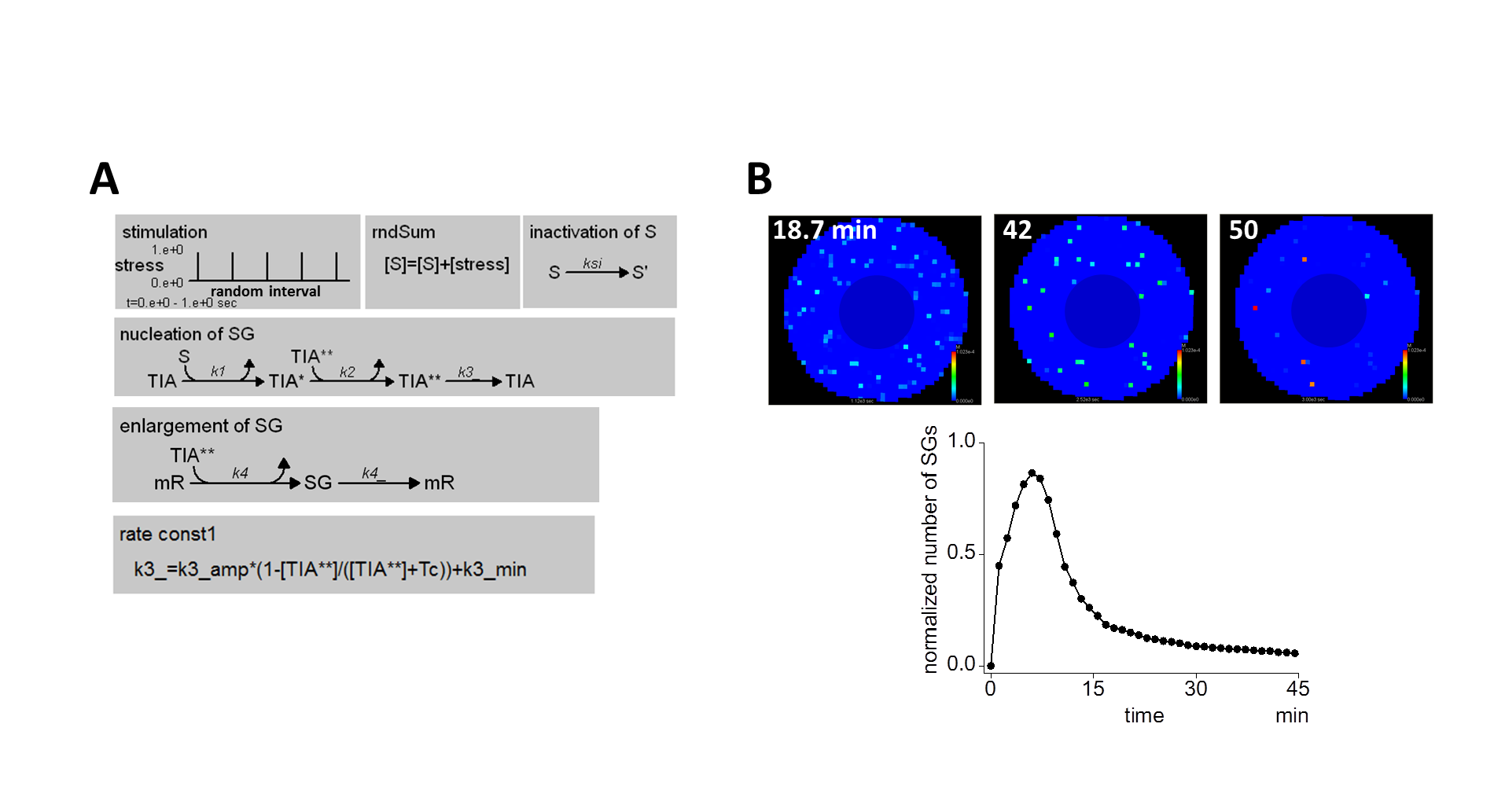

Supplement: S8 Fig — (A) We also tested DS model employing winners-share-all mechanism. Stress stimuli with random interval caused the formation of S in the DS model. S activated TIA, and activated TIA (TIA*) was further activated into TIA**, which is a positive feedback process. mRNA (mR) is assembled into SG by TIA**. S, TIA** and TIA** underwent self-inactivation. k 3_, which is a rate constant for TIA** inactivation, was assumed to be dependent on its concentration, which we assumed a measure of the size of a SG. These reaction schemes were embedded into 3D circular cell model (20 μm in diameter with a nucleus of 8.3 μm in diameter). (B) Simulation results of DS model were quantitatively agreed with our experiments (upper panels). However, there was no latency in the time course of SG assembly (lower panel). In addition, SGs could not be transported on microtubules, which did not agree with experimental observations. (TIF) [file pcbi.1004326.s013.tif]

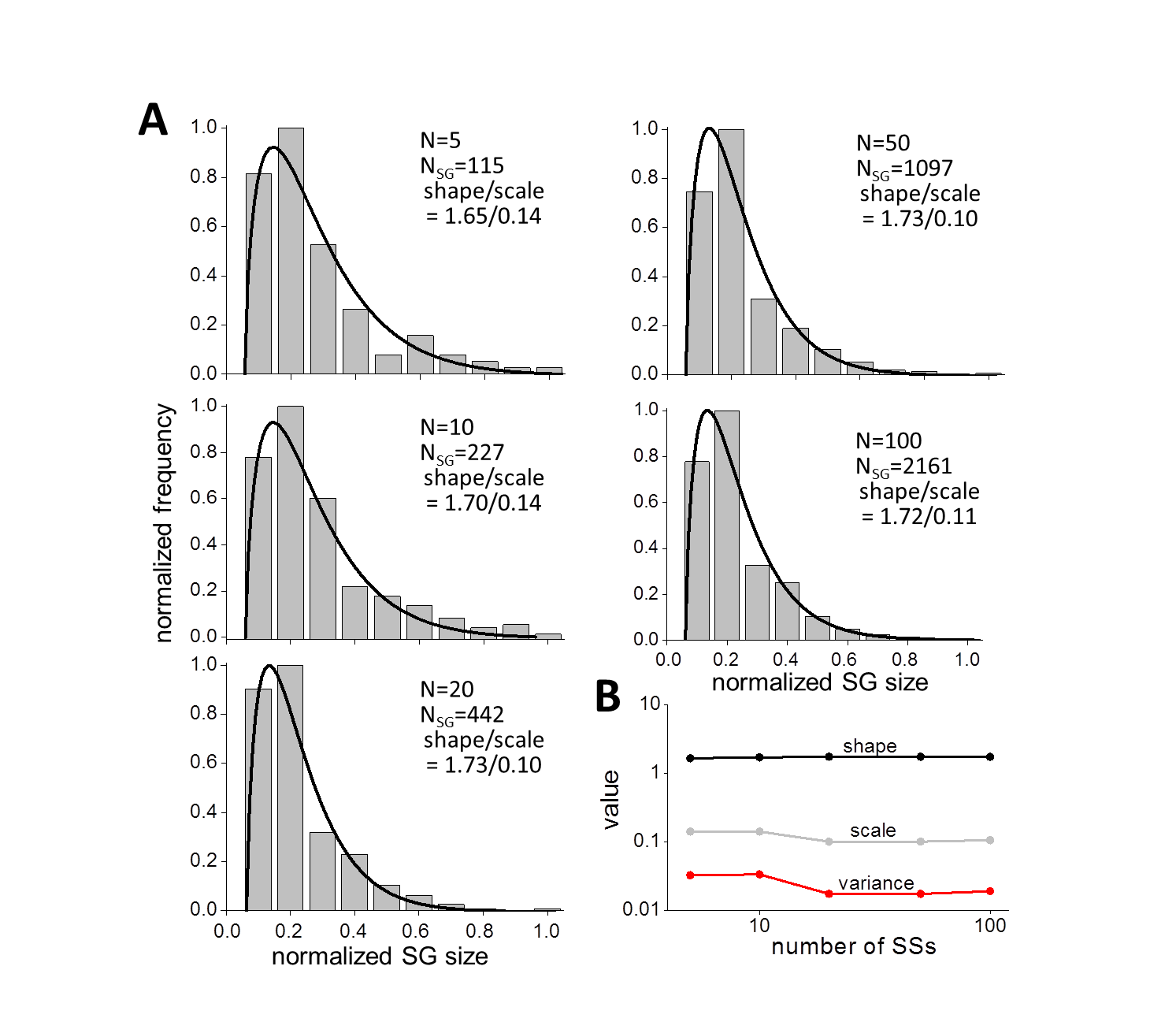

Supplement: S9 Fig — (A) We compared shape and scale parameters in different number of simulations of 5, 10, 20, 50, and 100. (B) The shape and scale parameters were almost unchanged. In addition, there was a small decrease in the variance, which was reasonable. (TIF) [file pcbi.1004326.s014.tif]

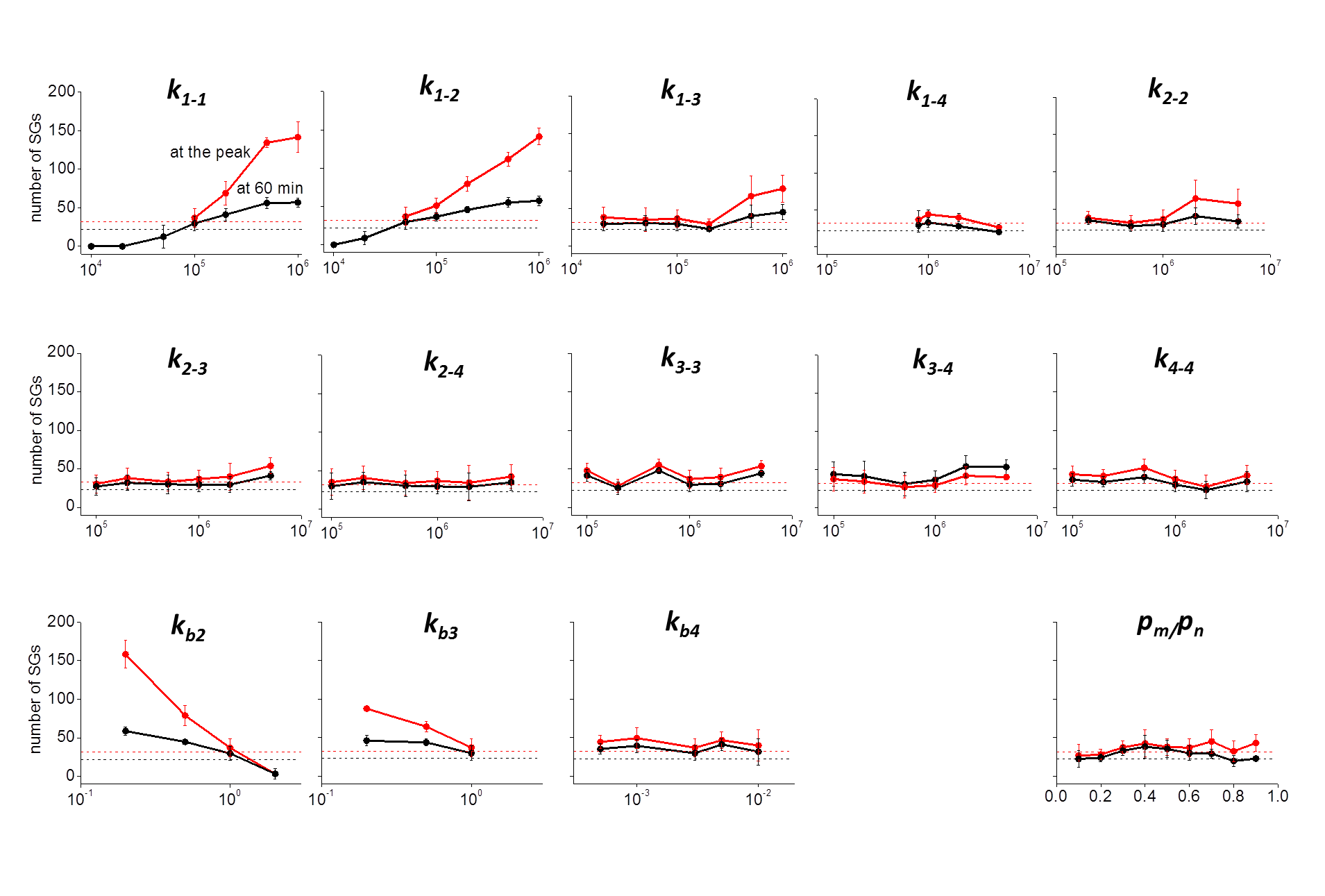

Supplement: S10 Fig — Forward and backward rate constants in the model of SG assembly were changed to see the change in the number of SG at the peak (red lines) and at 3600 sec (black lines). Red and black broken lines indicate data in the experiments for comparison. The changes in the SG number by the change in p m/p n are also shown. Canonical parameter values were as follows: k 1-1 = 105; k 1-2 = 5x10^4^; k 1-3 = 105; k 1-4 = 8x10^5^; k 2-2 = 106; k 2-3 = 106; k 2-4 = 106; k 3-3 = 106; k 3-4 = 106; k 4-4 = 106; k b2 = 1; k b3 = 0.1; k b4 = 3x10^-3^; p m = 0.4; p n = 0.6 (Units for forward and backward rate constants were /M/s and /s, respectively). One parameter was changed for the range shown in each graph leaving other parameters unchanged from canonical values. Missing data points, which are seen in the peak value at k 1-1 lower than 105 /M/s for example, indicate that there was no peak because of a big latency or no SG was assembled (Cf. S14 Fig). It is interesting to see that the difference between the peak value and that at 60 min increased as the increase in k 1-1 and k 1-2. See Materials and Methodsfor the calculation of sensitivity. (TIF) [file pcbi.1004326.s015.tif]

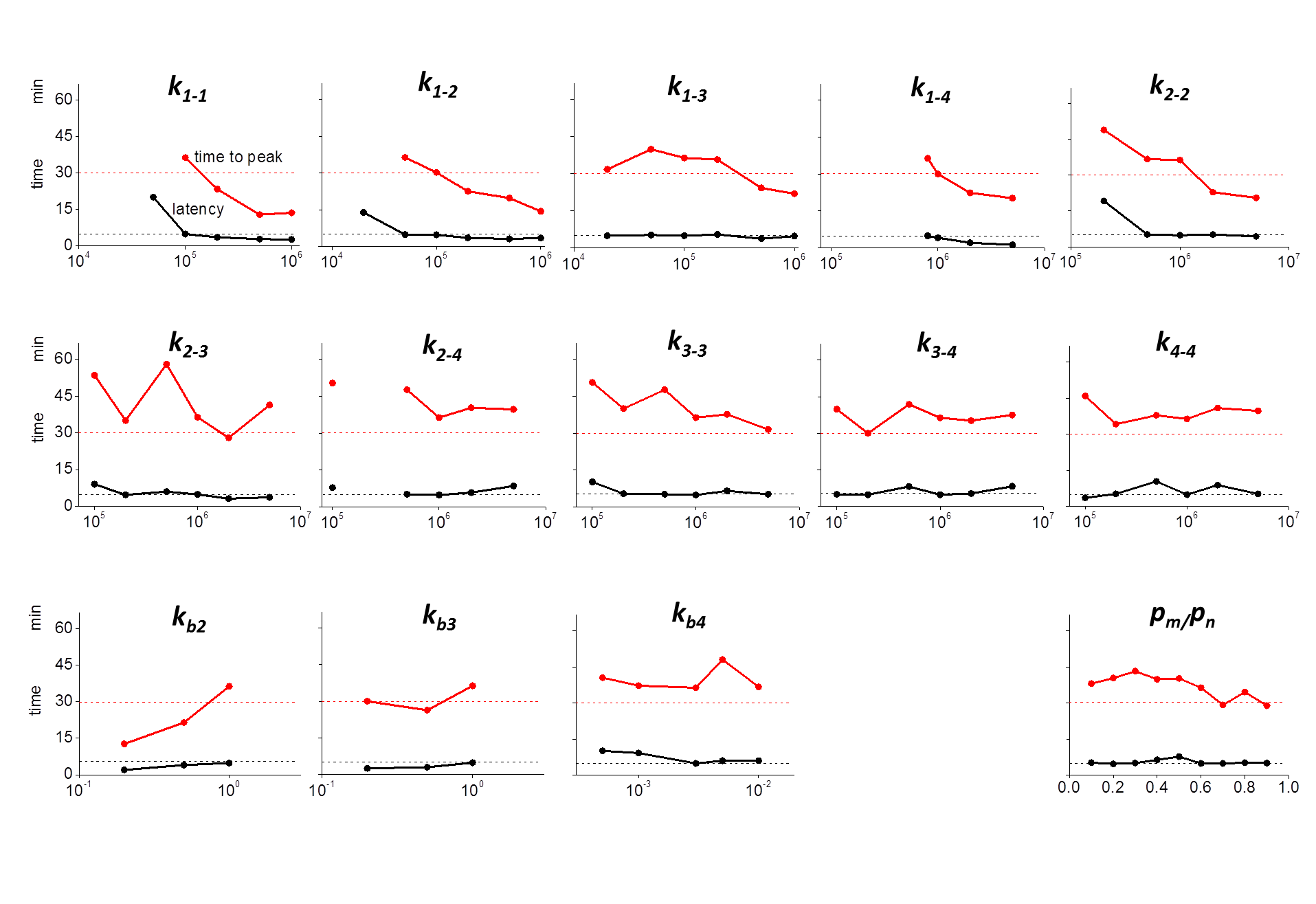

Supplement: S11 Fig — The sensitivity of the latency and the time to peak on kinetic parameters were tested as in S10 Fig. Forward and backward rate constants in the model for SG assembly were changed to see the change in the latency (black lines) and the time to peak (red lines). It is interesting to find that the latency was only sensitive to the smaller k 1-1, k 1-2, and k 2-2. Red and black broken lines show data from our experiments. Methods of calculating sensitivity was the same as in S10 Fig. (TIF) [file pcbi.1004326.s016.tif]

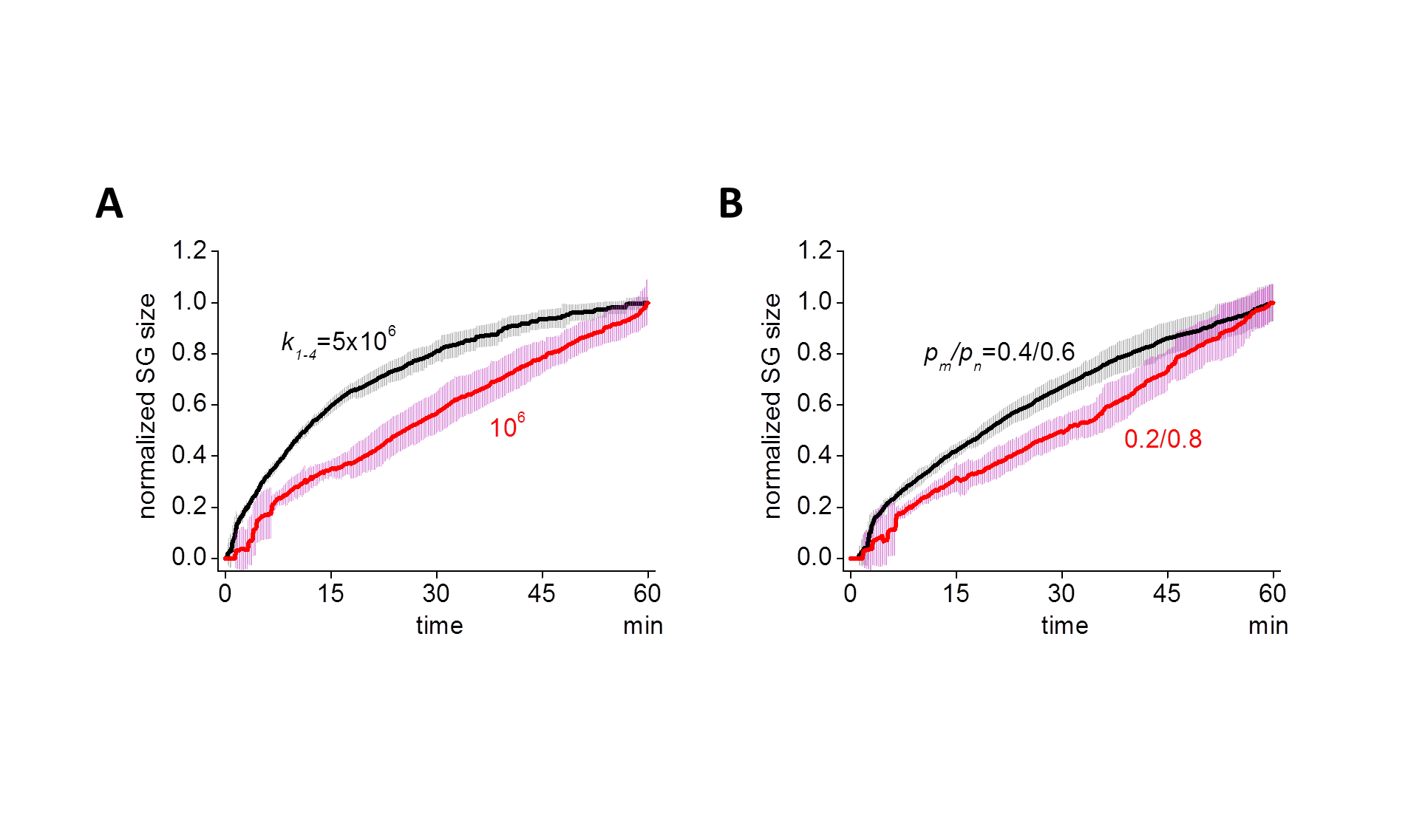

Supplement: S12 Fig — k 1-4 and p m/p n ratio changed the sublinearity. (TIF) [file pcbi.1004326.s017.tif]

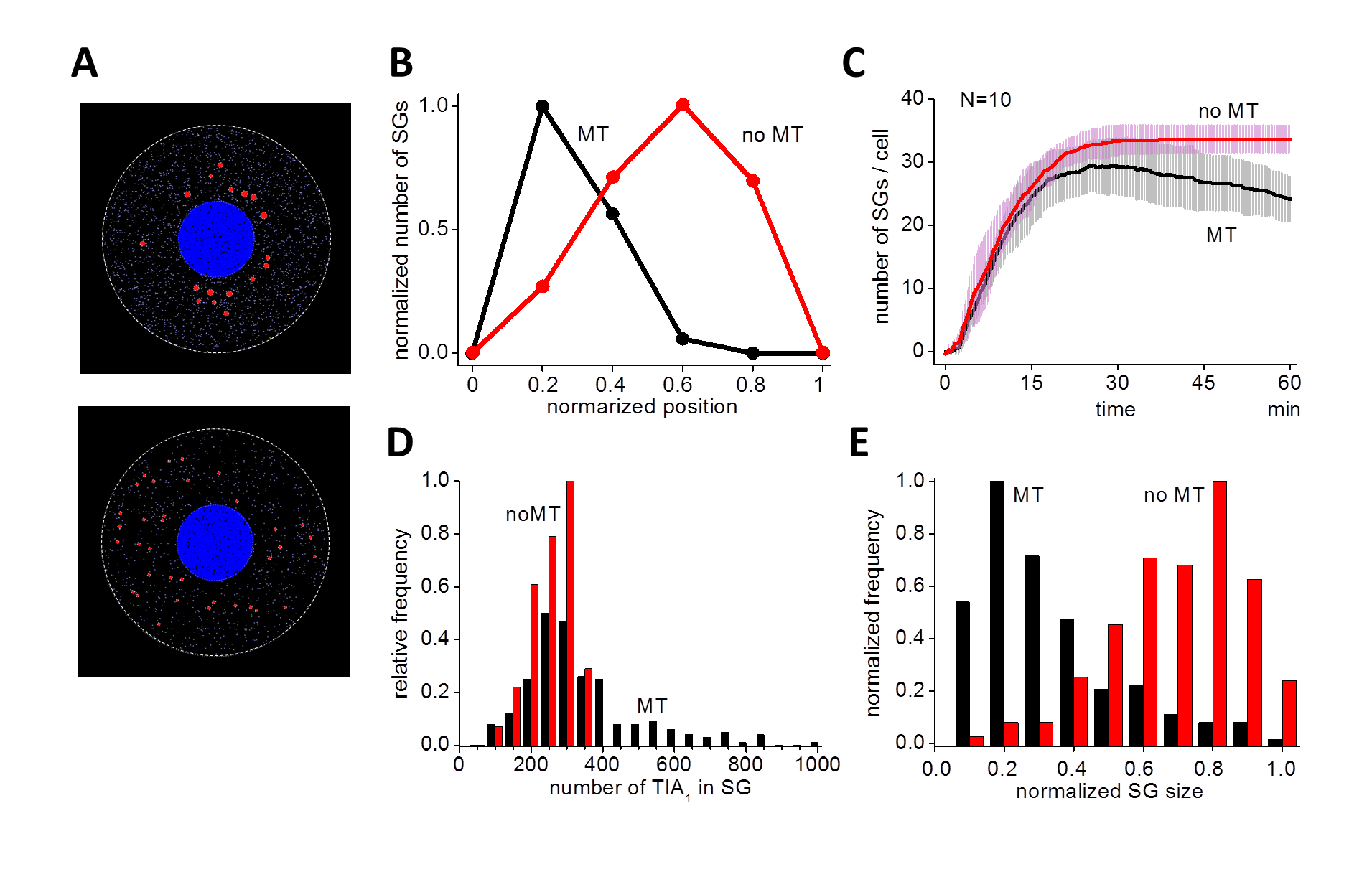

Supplement: S13 Fig — (A) SS results with (top panel) and without (bottom panel) microtubules at 60 min. In the absence of microtubules, small SGs were distributed throughout the cytoplasmic space. (B) Spatial distribution of SGs in the absence of microtubules (noMT, red line) was largely different from that in the presence of microtubules with p m/p n of 0.4/0.6 (MT, black line). (C) There was no decrease in the number of SGs during SG assembly in the absence of microtubules. (D) SG size distributed at smaller SG bins in the absence of microtubules (red bars). The histogram was normalized to the maximum frequency in the absence of microtubules. (E) While the SG size distribution skewed positively in the presence of microtubules (black bars), it skewed negatively in their absence (red bars). (TIF) [file pcbi.1004326.s018.tif]

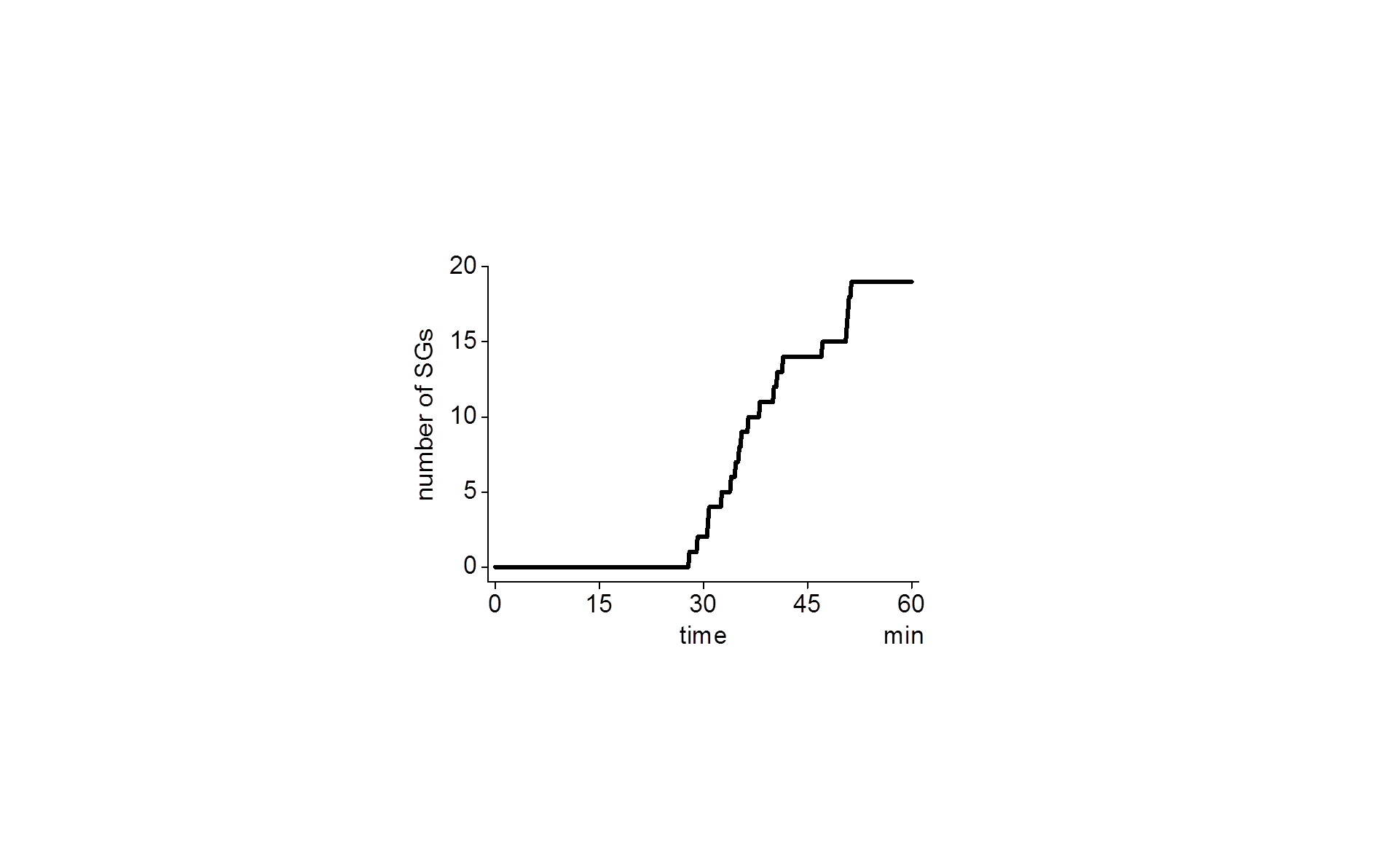

Supplement: S14 Fig — In case of long latency before the assembly of SGs, we could not observe a decrease in the number of SG during 60 min, and it was impossible to define the time to peak. (TIF) [file pcbi.1004326.s019.tif]

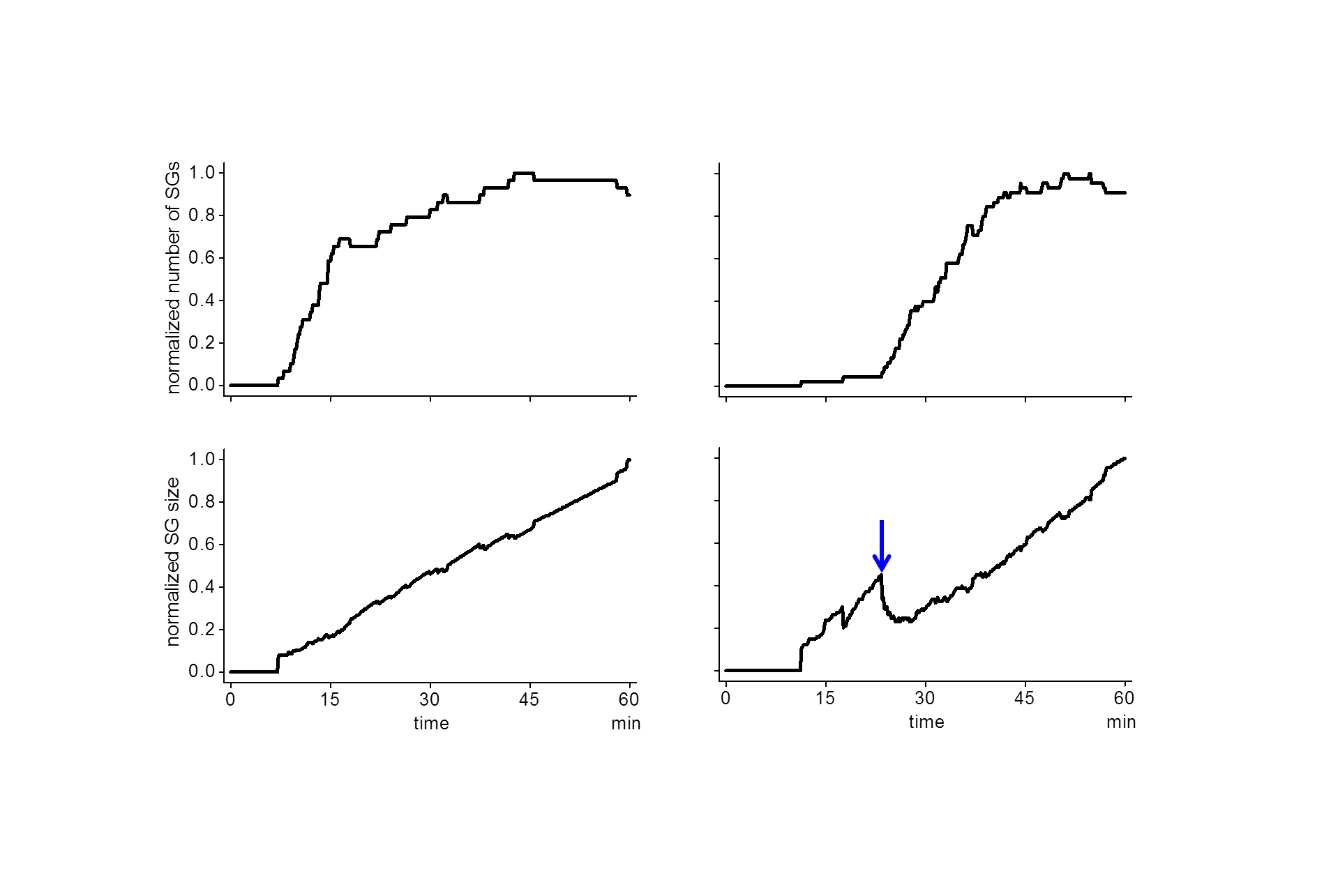

Supplement: S15 Fig — In many cases of long latency before SG assembly (right panels), we saw catastrophic disassembly of SGs (blue arrow). In contrast, there was no such catastrophic disassembly in a simulation with normal latency (left panels). (TIF) [file pcbi.1004326.s020.tif]
